# Differentiating sporadic frontotemporal dementia from late-onset primary psychiatric disorders

**DOI:** 10.1093/braincomms/fcaf199

**Published:** 2025-05-22

**Authors:** Sterre C M de Boer, Lina Riedl, Simon Braak, Chiara Fenoglio, David Foxe, James Carrick, Ramón Landin-Romero, Sophie Matis, Zac Chatterton, Ishana Rue, Marie-Paule E van Engelen, Jay L P Fieldhouse, Mardien Oudega, Sigfried N T M Schouws, Welmoed A Krudop, Argonde C van Harten, Flora H Duits, Sven J van der Lee, Daniela Galimberti, Janine Diehl-Schmid, Glenda M Halliday, Simon Ducharme, Yolande A L Pijnenburg, Olivier Piguet

**Affiliations:** Alzheimer Center Amsterdam, Department of Neurology, Vrije Universiteit Amsterdam, Amsterdam UMC Location VUmc, Amsterdam 1081HZ, The Netherlands; Amsterdam Neuroscience, Neurodegeneration, Amsterdam 1081HV, The Netherlands; Faculty of Science, School of Psychology, Brain and Mind Centre, The University of Sydney, Camperdown, NSW 2050, Australia; Department of Psychiatry and Psychotherapy, School of Medicine, Technical University of Munich, München 80333, Germany; Department of Psychiatry, Amsterdam UMC, Location Vrije Universiteit Amsterdam, Amsterdam 1081HV, The Netherlands; Amsterdam Neuroscience, Mood, Anxiety, Psychosis, Sleep & Stress and Neurodegeneration Programs, Amsterdam 1081HV, The Netherlands; Department of Biomedical, Surgical and Dental Sciences, University of Milan, Milano, MI 20133, Italy; Fondazione Ca’ Granda, IRCCS Ospedale Maggiore Policlinico, Milano, MI 20122, Italy; Faculty of Science, School of Psychology, Brain and Mind Centre, The University of Sydney, Camperdown, NSW 2050, Australia; Faculty of Science, School of Psychology, Brain and Mind Centre, The University of Sydney, Camperdown, NSW 2050, Australia; Faculty of Medicine and Health, School of Health Sciences, Brain and Mind Centre, The University of Sydney, Camperdown, NSW 2050, Australia; Faculty of Medicine and Health, School of Health Sciences, Brain and Mind Centre, The University of Sydney, Camperdown, NSW 2050, Australia; Faculty of Medicine and Health, School of Health Sciences, Brain and Mind Centre, The University of Sydney, Camperdown, NSW 2050, Australia; Department of Psychiatry, Douglas Mental Health University Institute, McGill University, Verdun, QC H4H 1R3, Canada; Alzheimer Center Amsterdam, Department of Neurology, Vrije Universiteit Amsterdam, Amsterdam UMC Location VUmc, Amsterdam 1081HZ, The Netherlands; Amsterdam Neuroscience, Neurodegeneration, Amsterdam 1081HV, The Netherlands; Alzheimer Center Amsterdam, Department of Neurology, Vrije Universiteit Amsterdam, Amsterdam UMC Location VUmc, Amsterdam 1081HZ, The Netherlands; Amsterdam Neuroscience, Neurodegeneration, Amsterdam 1081HV, The Netherlands; Alzheimer Center Amsterdam, Department of Neurology, Vrije Universiteit Amsterdam, Amsterdam UMC Location VUmc, Amsterdam 1081HZ, The Netherlands; Amsterdam Neuroscience, Neurodegeneration, Amsterdam 1081HV, The Netherlands; GGZ ingeest Amsterdam, Amsterdam 1081JC, The Netherlands; Alzheimer Center Amsterdam, Department of Neurology, Vrije Universiteit Amsterdam, Amsterdam UMC Location VUmc, Amsterdam 1081HZ, The Netherlands; Amsterdam Neuroscience, Neurodegeneration, Amsterdam 1081HV, The Netherlands; GGZ ingeest Amsterdam, Amsterdam 1081JC, The Netherlands; Alzheimer Center Amsterdam, Department of Neurology, Vrije Universiteit Amsterdam, Amsterdam UMC Location VUmc, Amsterdam 1081HZ, The Netherlands; Amsterdam Neuroscience, Neurodegeneration, Amsterdam 1081HV, The Netherlands; GGZ ingeest Amsterdam, Amsterdam 1081JC, The Netherlands; Alzheimer Center Amsterdam, Department of Neurology, Vrije Universiteit Amsterdam, Amsterdam UMC Location VUmc, Amsterdam 1081HZ, The Netherlands; Amsterdam Neuroscience, Neurodegeneration, Amsterdam 1081HV, The Netherlands; Alzheimer Center Amsterdam, Department of Neurology, Vrije Universiteit Amsterdam, Amsterdam UMC Location VUmc, Amsterdam 1081HZ, The Netherlands; Amsterdam Neuroscience, Neurodegeneration, Amsterdam 1081HV, The Netherlands; Alzheimer Center Amsterdam, Department of Neurology, Vrije Universiteit Amsterdam, Amsterdam UMC Location VUmc, Amsterdam 1081HZ, The Netherlands; Amsterdam Neuroscience, Neurodegeneration, Amsterdam 1081HV, The Netherlands; Genomics of Neurodegenerative Diseases and Aging, Department of Human Genetics, Vrije Universiteit Amsterdam, Amsterdam UMC Location VUmc, Amsterdam 1081HV, The Netherlands; Department of Biomedical, Surgical and Dental Sciences, University of Milan, Milano, MI 20133, Italy; Fondazione Ca’ Granda, IRCCS Ospedale Maggiore Policlinico, Milano, MI 20122, Italy; Department of Psychiatry and Psychotherapy, School of Medicine, Technical University of Munich, München 80333, Germany; Clinical Center for Psychiatry, Psychotherapy, Psychosomatic Medicine, Geriatrics and Neurology, kbo-Inn-Salzach-Klinikum, Wasserburg am Inn 83512, Germany; Faculty of Medicine and Health, School of Health Sciences, Brain and Mind Centre, The University of Sydney, Camperdown, NSW 2050, Australia; Department of Psychiatry, Douglas Mental Health University Institute, McGill University, Verdun, QC H4H 1R3, Canada; McConnell Brain Imaging Centre, Montreal Neurological Institute, McGill University, Montréal, QC H3A 2B4, Canada; Alzheimer Center Amsterdam, Department of Neurology, Vrije Universiteit Amsterdam, Amsterdam UMC Location VUmc, Amsterdam 1081HZ, The Netherlands; Amsterdam Neuroscience, Neurodegeneration, Amsterdam 1081HV, The Netherlands; Faculty of Science, School of Psychology, Brain and Mind Centre, The University of Sydney, Camperdown, NSW 2050, Australia

**Keywords:** frontotemporal dementia, psychiatry, diagnostics

## Abstract

Sporadic behavioural variant frontotemporal dementia (bvFTD) is often misdiagnosed as late-onset primary psychiatric disorder (PPD) due to overlapping symptoms and lack of biomarkers. We aimed to identify clinical features that distinguish sporadic bvFTD from PPD. Multi-centre baseline data were retrospectively retrieved and categorized into neuropsychological domains. Logistic regression models and receiver operating characteristic curves were conducted to determine discriminators. Data from 508 sporadic bvFTD and 152 PPD cases were included. Higher scores in cognitive screening [odds ratio (OR): 1.23], facial emotion processing (OR: 1.69), episodic memory (OR: 1.09), animal fluency (OR: 1.17), working memory (OR: 1.18), letter fluency (OR: 1.17) and depressive symptoms (OR: 7.41) were significantly associated with PPD (all *P*s ≤ 0.010). Within a combined model, higher scores of letter fluency (OR: 1.47), cognitive screening (OR: 1.72) and lower attention (OR: 0.77) were significantly (all *P*s ≤ 0.05) associated with PPD (area under the curve = 0.771). Neuropsychological measurements—letter fluency, cognitive screening and attention—can help distinguish sporadic bvFTD from late-onset PPD. Depressive symptoms and facial emotion processing emerged as potential discriminators, warranting further exploration.

## Teaser

de Boer *et al*. aimed to identify which neuropsychological and neuropsychiatric features measured at baseline visit had the strongest discriminative value to distinguish between sporadic behavioural variant frontotemporal dementia and late-onset primary psychiatric disorders. In this retrospective study, letter fluency, cognitive screening and attention were identified as the strongest clinical discriminators.

## Graphical Abstract

**Graphical Abstract:**
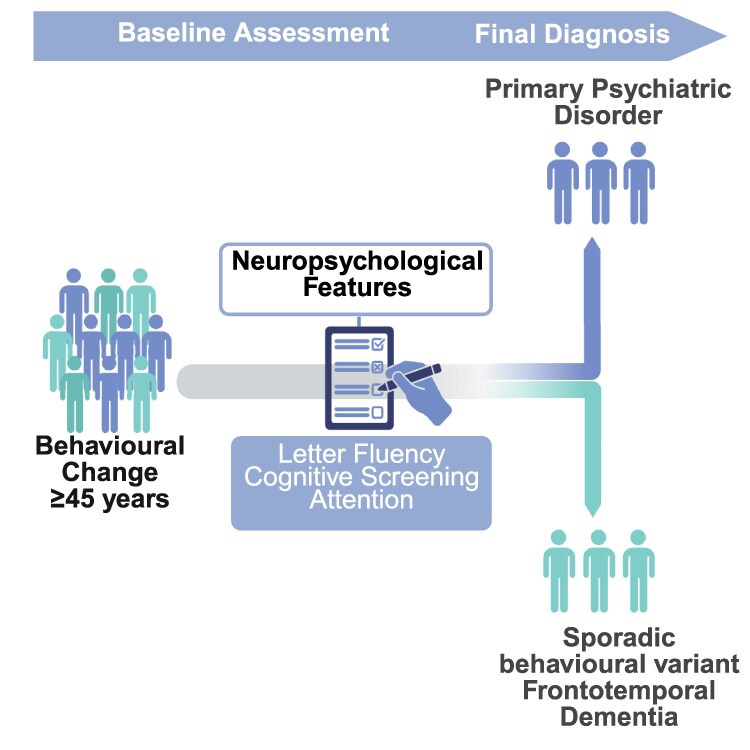

## Introduction

The behavioural variant frontotemporal dementia (bvFTD) is a progressive neurodegenerative brain disorder that affects the frontal and temporal lobe resulting in behavioural and personality changes, language impairment and executive dysfunction.^1-3^ The peak age at onset of bvFTD is between 40 and 60 years of age, making it the second most common cause of young onset dementia after Alzheimer’s disease.^4^ Between 20 and 30% of bvFTD cases exhibit a strong family history of the disease across multiple generations, mainly accounted for by the following genetic abnormalities: CCCCGG hexanucleotide repeat expansion in the non-coding region of chromosome 9 open reading frame 72 gene (*C9orf72*), mutations in the progranulin (*GRN*) or microtubule-associated protein tau (*MAPT*) genes.^5,6^ Most of the remaining cases of bvFTD lack identifiable family history and/or mono-genetic causes and are labelled sporadic bvFTD.^7^ Although they account for the majority of cases, sporadic bvFTD has received little attention compared with genetic bvFTD in previous research.^8^

bvFTD symptomatology overlaps with that found in primary psychiatric disorders (PPD), including major depressive disorder, bipolar disorder, schizophrenia, obsessive-compulsive disorder and personality disorders,^9^ making PPD the most common differential diagnosis for bvFTD.^10^ In addition to symptomatic similarity, the lack of a family history of FTD and genetic diagnostic markers makes an accurate diagnosis of sporadic bvFTD challenging, particularly in the early disease stages. Notably, between a quarter to half of patients are misdiagnosed,^11-13^ accompanied by lengthy delays (3–6 years) between symptom onset and diagnosis.^14,15^ This uncertainty results in a high psychosocial burden for the patients and caregivers and delays access to appropriate treatment options and interventions, an increasingly important issue in the light of current developments of therapeutics and disease-modifying treatments in FTD.^16^

We have established the international multi-centre ‘Diagnostic and Prognostic Precision Algorithm FTD’ (DIPPA-FTD) project in order to improve the differentiation of sporadic bvFTD from PPD.^17^ DIPPA-FTD, which aims to develop diagnostic and prognostic tools to distinguish sporadic bvFTD from late-onset PPD in its earliest stages, consists of two arms: a retrospective cohort study and a prospective study. The retrospective cohort combines clinical, neuropsychological and social cognition markers, structural neuroimaging, blood-based biomarkers and pathological data sets from five international centres specialized in neuropsychiatry and dementia. Here, this study focused on the clinical components of the retrospective cohort and aimed to identify the clinical and neuropsychological indices that most robustly differentiate sporadic bvFTD from late-onset PPD. Findings from the retrospective analyses will eventually inform the design of the prospective arm of the DIPPA-FTD research programme.

## Materials and methods

### Study structure

DIPPA-FTD is an international multi-centre retrospective observational cohort study from the Alzheimer Center Amsterdam, the Brain and Mind Centre at the University of Sydney, the Douglas Mental Health University Institute at McGill University, Fondazione Ca’ Granda, IRCCS Ospedale Maggiore Policlinico, University of Milan and the Technical University of Munich. The study was approved by local ethical committees. No participants were excluded on the basis of their gender, ethnicity or cultural background. However, legal requirements regarding the informed consent to share personal data for retrospective data analyses prevented access to ethnic background information, thereby preventing quantification of representation relative to population estimates. It is possible that some minorities may be underrepresented.

### Participants

The retrospective DIPPA-FTD study included individuals aged 45 years and older presenting with behavioural change, who were eventually diagnosed with either sporadic bvFTD or with a late-onset PPD meeting one of the Diagnostic and Statistical Manual of Mental Disorders, fifth edition classifications for major depressive disorder (MDD), manic episode, bipolar disorder, schizophrenia, personality disorders, delusional disorder or obsessive-compulsive disorder.^18^ Individuals with a prior psychiatric history before age 45 were eligible for inclusion if their early-life psychiatric history was unrelated to the current late-onset behavioural change, such as a patient with late-onset delusional disorder who experienced a mild depressive episode as adolescent after a major life event. Patients with bvFTD met current diagnostic criteria for probable or definite bvFTD (based on pathology)^2^ with a Clinical Dementia Rating^19^ score of ≤1. Diagnoses were established according to the International Consensus Diagnostic Criteria for bvFTD^2^ or Diagnostic and Statistical Manual of Mental Disorders, fifth edition.^18^ Diagnoses were made by neurologists, psychiatrists or geriatricians, specialized in FTD and/or PPD, operating within the participating centres. Clinical diagnostic workup followed local guidelines in each of the participating centres, involving measures such as (informant-based) history-taking, neuropsychological and neuropsychiatric assessments and brain imaging. To minimize the risk of including an individual with a genetic form of FTD, cases were classified using Goldman^20-22^ or Wood scores.^23^ A Goldman score of 1 indicates a strong family history of FTLD whereas the highest score of 4 reflects an absence of family history for FTLD or that the family history is unknown. Wood scores range from ‘high’ to ‘apparent sporadic’. Here, a case was classified as sporadic bvFTD on the condition of having a Goldman score of ≥3 or a Wood score of ‘low’ or ‘apparent sporadic’ in the absence of a *C9orf72* repeat expansion. Participants with a Goldman score of 2 or a Wood score of ‘medium’ were included if genetic testing of (at least) *C9orf72*, *GRN* and *MAPT*, the three most common pathogenic genetic abnormalities in FTD at each participating site, was negative. Individuals with a Goldman score of 1 and Wood score ‘high’ were excluded, as these scores are likely to be associated with pathogenic mutations. Individuals diagnosed with PPD were only included if the clinicians were certain of the diagnosis and if screening for the presence of *C9orf72* repeat expansion was negative.

All participants provided informed consent to participate in the original local studies and to share their data for future research purposes. The study was approved by local ethical committees and was performed in accordance with the ethical standards as laid down in the 1964 Declaration of Helsinki and its later amendments.

### Clinical data harmonization methods

Each site collected clinical demographics, neuropsychological and social cognition tests, neuroimaging and blood for their local biobank(s) at baseline and at follow-up visits. In the present study, clinical demographics and neuropsychological data acquired within a 6-month window of the first visit were included and classified into eight domains: (I) global cognitive screening, (II) facial emotion processing, (III) episodic memory, (IV) animal fluency, (V) attention, (VI) working memory, (VII) letter fluency and (VIII) neuropsychiatric symptoms. As anticipated, clinical assessment and protocols differed across sites, leading to a heterogeneous data set with variable percentages of missing data. To overcome this issue, we applied four data harmonization methods in order to maximize data combinations across sites, as follows:

- ‘raw scores’ were used when test protocols across sites were identical;
- ‘conversion’ was applied to convert results from different tests measuring the same construct into a common metric using published methods;
- ‘min–max scaling’ was used to transform data into one range (0–1) by using the min–max method (xm = (x − xmin)/(xmax − xmin)).^24^ Prior to min–max scaling, outliers (i.e. ±3 SD from the group mean) were removed. Outlier thresholds were calculated for PPD and FTD separately; and
- ‘dichotomizing’ method converted continuous variables into a dichotomous variable by predefined and validated cut-off points.

### Neuropsychological domains and harmonization

Following examination of data availability in the different sites, we selected the cognitive measures that had the smallest number of missing values within sites and had the largest amount of data points available across sites. These measures were selected for harmonization and analyses. There were different reasons for missing values; either data were not available (i.e. task not administered in that site), or participant(s) did not complete the task. If multiple measures of a particular test were available (e.g. letters ‘D’ and ‘S’ for letter fluency), an average between these measures was calculated. All tests harmonized into one domain showed similar distributions.

**Figure 1:**
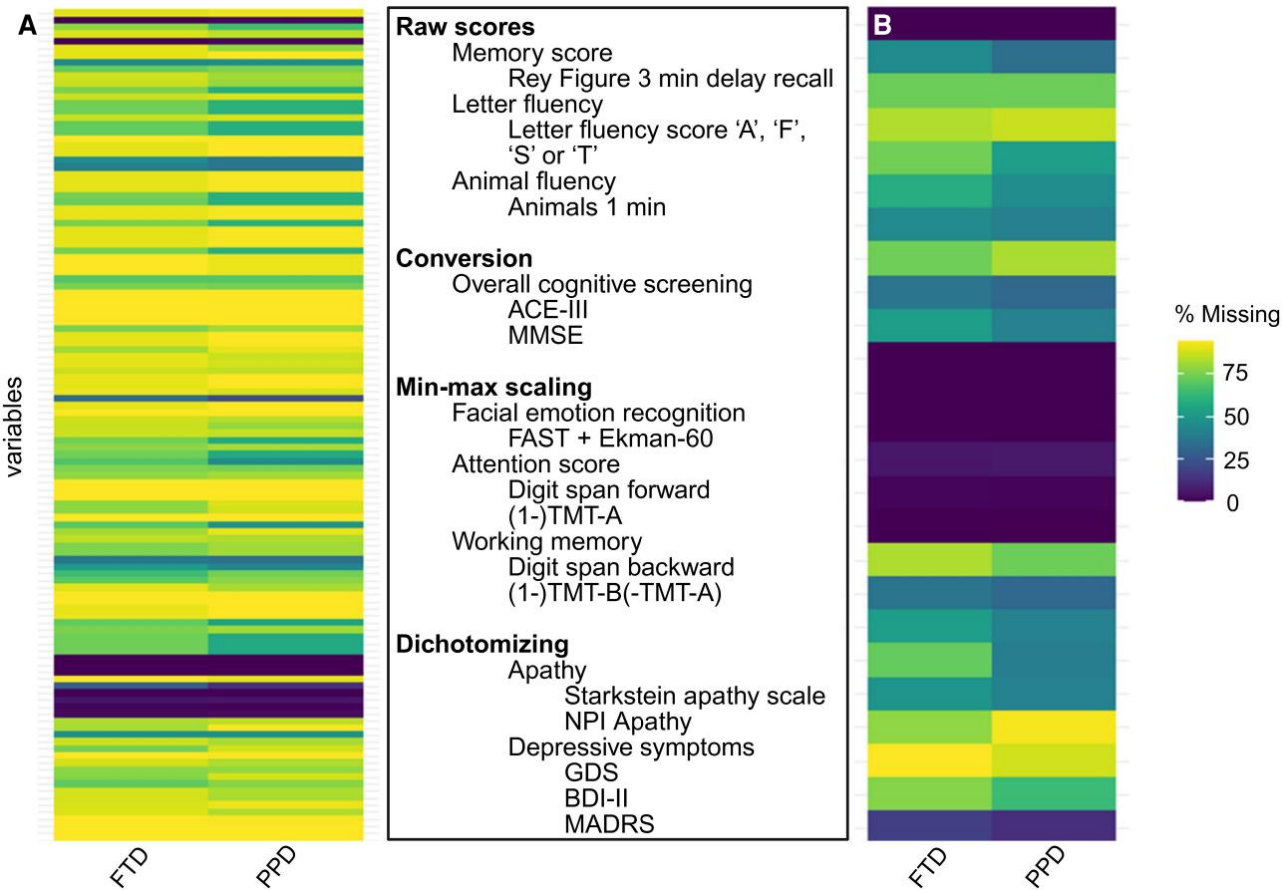
**Visual illustration of missing data before (left) and after (right) data harmonization.** The *x*-axis in the heatmap represents a subset % of missing data for each variable. *y*-axis represents the diagnostic group (bvFTD and PPD). ACE-III, Addenbrooke’s Cognitive Examination III; BDI-II, Beck Depression Inventory II; FAST, Facial Affect Selection Test; FTD, frontotemporal dementia; GDS, Geriatric Depression Scale; MADRS, Montgomery Åsberg Depression Rating Scale; MMSE, Mini-Mental State Examination; NPI, Neuropsychiatric Inventory; PPD, primary psychiatric disorders; TMT, Trail Making Test. Visual illustration created with Biorender.com.

The global cognitive screening domain (I) included the Mini-Mental State Examination (MMSE)^25^ or the Addenbrooke's Cognitive Examination-III (ACE-III).^26^ ACE-III scores were converted to MMSE scores as previously published.^27^ The facial emotion processing domain (II) included the Facial Affect Selection Task^28,29^ and the Ekman-60 Faces test.^30^ These scores were harmonized by min–max scaling. No transformation was needed for the episodic memory domain (III) where the raw score from the Rey Complex Figure test 3-min delayed recall was used.^31,32^ Likewise, animal fluency was used to measure the semantic fluency domain (IV) and comprised the raw score from the ‘animal fluency 1 min’. The attention domain (V) was obtained by min–max scaling and harmonizing the Digit Span Forward^33^ and Trail Making Test Part A (TMT-A)^34^ measures. The min–max scaled score for TMT-A was further inverted (i.e. 1 minus TMT-A score) as a higher score on this task indicates worse performance, unlike Digit Span Forward where a higher score reflects better performance. Similarly, the working memory domain (VI) was obtained by min–max scaling and harmonizing the Digit Span Backward and inverting the TMT-B^35^ minus TMT-A score. Letter fluency was used as a measure of executive function (VII) and was obtained by averaging the raw scores of the letter fluency task for the letters ‘A’, ‘F’, ‘S’ or ‘T’.^36^ For the neuropsychiatric domain, two independent scores were extracted. The first score reflected depressive symptomatology, which was derived by dichotomizing in ‘absent’ or ‘present’ scores from the Geriatric Depression Scale −15 item version^37,38^ (‘absent’: <6, ‘present’: ≥6), the Beck Depression Scale version II^39,40^ (‘absent’: <14, ‘present’: ≥14) or the Montgomery Åsberg Depression Rating Scale^41^ (‘absent’: <7, ‘present’: ≥7). The second score reflected the presence or absence of apathy symptoms by dichotomizing scores of the Starkstein Apathy Scale^42^ (‘absent’: <14, ‘present’: ≥14) or the Neuropsychiatric Inventory^43,44^ question on apathy (‘yes’ or ‘no’). A visual representation of missing data in each clinical group and the harmonization methods for each of neuropsychological domain is shown in Fig. 1. Additional methodological information on the harmonization methods used for domains (II), (V) and (VI) can be found in Supplementary Methods 1. We acknowledge that different ways of categorizing and combining the available measurements in cognitive constructs are possible. Although grounded in solid theoretical considerations, we recognize that our method to categorize these measurements may not be the only possible approach.

### Statistical analyses

All statistical analyses were performed using R (version 4.2.1, R Development Core Team, 2020). Missing data heatmaps were created using the naniar package.^45^ Baseline demographics and neuropsychological scores were compared between bvFTD and PPD. Normally distributed continuous values were compared using *t*-tests. Non-normally distributed continuous values were examined using Mann–Whitney *U* tests. Pearson's *χ*^2^ test was used when dichotomous variables were compared. To correct for multiple comparisons, results were considered significant if *P* < 0.0055 (*P* 0.05 divided by the nine logistic regression models that were run).

To assess diagnostic classification strength of each domain, we performed logistic regression models, corrected for age (at time of assessment), sex and education (years), for each domain. Missing data were imputed for education using site-specific mean imputation. The reference group was set to sporadic bvFTD. Interpretability of the min–max-scaled variables was improved by multiplying the scores with 10 so that one-unit increase in test score equals a 10% increase of correct score. In order to maximize available data points, the regression models were performed separately for each domain using subsets of data with no missing values in the variables under consideration for that particular domain. As such, the number of cases included in each model differed and prevented direct comparisons across the models.

Finally, we performed one overall logistic regression analysis that combined all the variables, corrected for age, sex and education, using the most optimal (e.g. no missing data) data set to identify the best discriminator(s) between bvFTD and PPD. For the latter analysis, all variables were converted to a min–max-scaled score, enabling comparing odds ratios (OR) between the neuropsychological domains. Receiver operating characteristic (ROC) curves were plotted and area under the curve (AUC) calculated, using the pROC R-package,^46^ to identify the best model and clinical discriminator.

## Results

Clinical data from baseline visit from a total of 660 patients (bvFTD = 508; PPD = 152) were included. Diagnostic characteristics of the study samples are shown in Table 1.

**Table 1:** Diagnosis per each diagnostic group

Diagnostic group	Diagnosis	*n*
FTD	Probable bvFTD	492
	Definite bvFTD	16
PPD	Major depressive disorder	107
	Bipolar disorder	18
	Delusional disorder	6
	Schizophrenia	4
	Schizoaffective disorder	3
	Manic episode	2
	Obsessive compulsive disorder	1
	Psychiatric disorders NOS^a^	11
Total		660

bvFTD, behavioural variant frontotemporal dementia; NOS, not otherwise specified; PPD, primary psychiatric disorder.

^a^Participants diagnosed with a psychiatric disorder but not further classified into one of the Diagnostic and Statistical Manual of Mental Disorders, fifth edition categories.

Distributions of the diagnoses per centre can be found in Supplementary Material 1. Patient characteristics for the sporadic bvFTD and PPD group are shown in Table 2. PPD patients were significantly younger than bvFTD patients at both time points (symptom onset, age at first visit, both *P*s < 0.001). Among the study participants, 230/508 (45.3%) had Alzheimer’s disease biomarkers available (e.g. amyloid PET scan or determination of amyloid-beta and p-tau in cerebrospinal fluid). Within the bvFTD group, 17/183 (9.3%) were amyloid- and tau-positive, compared with 3/47 (6.4%) within the PPD group. Sex distribution between the bvFTD and PPD and education years did not significantly differ between groups.

**Table 2:** Demographic and clinical characteristics of sporadic bvFTD and PPD participants

	bvFTD	PPD	*P*-value
***n* (% total)**	508 (77.0)	152 (23.0)	n.a.
**Sex, % male**	58.7	65.1	0.18^a^
**Education, mean years (SD)**	11.7 (3.9)	11.6 (3.5)	0.83^b^
**Age at onset, mean (SD)**	61.5 (9.1)	55.3 (8.1)	<0.001^b^
**Age at first visit, median (IQR)**	65.0 (12.0)	59.0 (10.5)	<0.001^c^
**Age at neuropsychology tests, median (IQR)**	65.0 (13.0)	59.0 (12.0)	<0.001^c^
**Disease duration, median years (IQR)**	2.5 (2.9)	3.0 (3.6)	0.68^c^
**Deceased, *n***	148	8	n.a.
**Mean age at death (SD)**	67.2 (8.4)	65.5 (12.1)	n.a.
**Pathology available, *n***	16^d^	0	n.a.

bvFTD, behavioural variant frontotemporal dementia; IQR, interquartile range; PPD, primary psychiatric disorder; SD, standard deviation.

^a^Chi-square.

^b^ *t*-test.

^c^Mann–Whitney *U*.

^d^Pathology subtypes: FUS *n* = 2, TAU *n* = 8, TDP *n* = 5 and FTLD other *n* = 1.

Harmonization methods, data availability per diagnostic group and average scores for each domain are shown in Table 3. The FTD group had significantly lower scores than the PPD group for the domains global cognitive screening, facial emotion processing, episodic memory score, animal fluency, letter fluency and depressive symptoms (all *P*s < 0.0055; Table 3). Scores for the domains of attention, working memory and apathy did not significantly differ between bvFTD and PPD (*P* = 0.04, *P* = 0.04 and *P* = 1.00, respectively; Table 3). Average scores for each domain per diagnostic subtype in the PPD group can be found in Supplementary Material 2.

**Table 3:** Performance on neuropsychological and neuropsychiatry domains in sporadic bvFTD and PPD

Neuropsychological and neuropsychiatry domains	Harmonization method	bvFTD (*n*)/PPD (*n*)	bvFTD	PPD	*P*-value
Global cognitive screening, median (IQR)	(b)	419/133	26.0 (4.0)	28.0 (4.0)	2.01e^−12^c^^
Facial emotion processing, median (IQR)	(c)	132/26	0.68 (0.3)	0.80 (0.2)	1.17e^−03^c^^
Episodic memory score, median (IQR)	(a)	129/40	11.0 (10.5)	17.0 (8.7)	1.79e^−04^c^^
Animal fluency, mean (SD)	(a)	217/82	11.9 (6.3)	18.2 (5.8)	7.21e^−14^b^^
Attention, mean (SD)	(c)	387/118	0.55 (0.2)	0.59 (0.2)	0.04^b^
Working memory, mean (SD)	(c)	278/100	0.39 (0.2)	0.43 (0.2)	0.04^b^
Letter fluency, median (IQR)	(a)	257/89	7.2 (6.2)	11 (6.7)	1.62e^−08^c^^
NPS—depressive symptoms, present (%)	(d)	140/91	24.3	71.4	3.96e^−12^a^^
NPS—apathy, present (%)	(d)	115/54	73.9	74.1	1.00^a^

Harmonization methods: (a) raw scores, (b) conversion, (c) min–max scaling and (d) dichotomizing.

bvFTD, behavioural variant frontotemporal dementia; IQR, interquartile range; NPS, neuropsychiatric symptoms; PPD, primary psychiatric disorders; SD, standard deviation.

^a^Chi-square.

^b^ *t*-test.

^c^Mann–Whitney *U*.

As indicated in the Materials and methods section, logistic regression analyses were carried out on each domain separately. Each model included age, sex and education as covariates. These analyses showed that higher scores in the domains global cognitive screening (OR 1.23, *P* < 0.001), facial emotion processing (OR 1.69, *P* < 0.001), episodic memory score (OR 1.09, *P* < 0.001), animal fluency (OR 1.17, *P* < 0.001), working memory (OR 1.18, *P* = 0.010), letter fluency (OR 1.17, *P* < 0.001) and presence of depressive symptoms (OR 7.41, *P* < 0.001) were all significantly associated with an increased likelihood of PPD (Table 4). In contrast, attention score (*P* = 0.130) and the presence of apathy symptoms (*P* = 0.640) were not significantly associated with group membership. Output data from each comparison and logistic regression can be found in Supplementary Material 3.

**Table 4:** Logistic regression of each domain per subset

Domain	Estimate	*P*-value	OR	95% CI
Global cognitive screening	0.21	<0.001	1.23	1.14, 1.33
Facial emotion processing	0.52	<0.001	1.69	1.26, 2.40
Episodic memory score	0.08	<0.001	1.09	1.03, 1.15
Animal fluency	0.16	<0.001	1.17	1.11, 1.23
Attention score	0.09	0.130	1.10	0.97, 1.24
Working memory	0.17	0.010	1.18	1.05, 1.34
Letter fluency	0.16	<0.001	1.17	1.11, 1.25
NPS—depressive symptoms	2.00	<0.001	7.41	3.97, 14.27
NPS—apathy	−0.19	0.640	0.82	0.37, 1.86

Reference group is bvFTD. All regression analyses are corrected for age, sex and education. Each row represents a separate logistic regression analysis in a subset of the total cohort. See Table 3 Column 3 for number of cases included in each model. See Supplementary Material 2 for output of all separate models.

CI, confidence interval; NPS, neuropsychiatric symptoms; OR, odds ratio.

A final analysis was conducted by combining the domains with the largest number of cases with no missing data. This maximal and optimal data set contained 292 cases (*n* = 217 bvFTD, *n* = 75 PPD; see breakdown of PPD diagnosis in Supplementary Material 4) with data from four domains: global cognitive screening, attention, working memory and letter fluency. To facilitate OR comparisons across domains in the model, all domain scores were min–max scaled if not already scaled. Logistic regression analysis in the optimal data set, corrected for age, sex and education, showed that 10% increases in scores resulted in a higher odds of PPD classification for global cognitive screening (OR 1.72, *P* = 0.008) and letter fluency (OR 1.47, *P* < 0.001), but a lower odds of being classified as PPD for attention (OR 0.77, *P* = 0.049; Table 5). Working memory was not found to contribute significantly to the model (*P* = 0.498). The diagnostic subtypes of the PPD group that were included in the optimal data set can be found in Supplementary Table 4.

**Table 5:** Logistic regression model of optimal data set

	Estimate	*P*-value	OR	95% CI
Intercept	−0.25	0.900	0.78	0.01, 41.34
Global cognitive screening	0.54	0.008	1.72	1.18, 2.62
Attention	−0.27	0.049	0.77	0.58, 0.99
Working memory	0.07	0.498	1.07	0.87, 1.32
Letter fluency	0.39	<0.001	1.47	1.21, 1.80

Corrected for age, education and sex. All variables are min–max scaled. One-unit increase represents 10% increase in correct score for each variable. Reference group: bvFTD. Number of cases included in the model: bvFTD *n* = 217, PPD *n* = 75.

bvFTD, behavioural variant frontotemporal dementia; CI, confidence interval; OR, odds ratio; PPD, primary psychiatric disorder.

**Figure 2:**
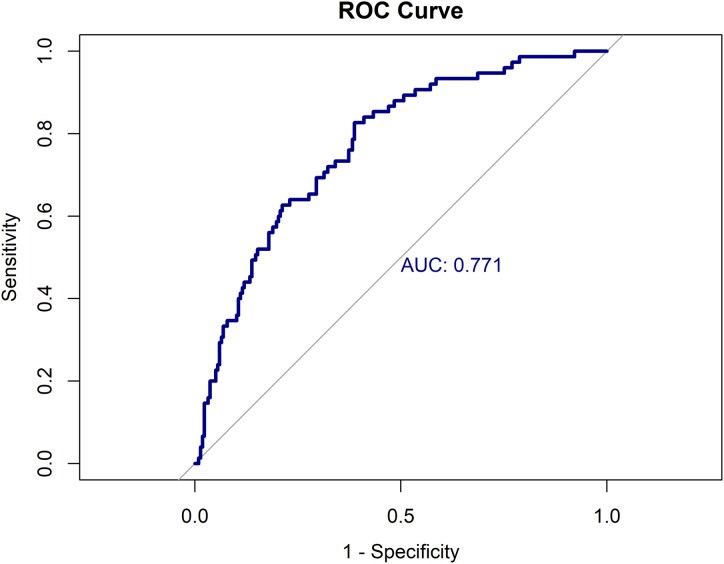
**ROC curves and AUC of most optimal model to distinguish sporadic bvFTD from PPD (*n* = 292 cases, *n* = 217 bvFTD, *n* = 75 PPD).** ROC curve of model including global cognitive screening, attention, working memory and letter fluency, corrected for age, sex and education. AUC, area under the curve; ROC, receiver operating characteristic.

Based on the regression model that included global cognitive screening, attention, working memory and letter fluency, corrected for age, sex and education, the ROC curve yielded an AUC of 0.771 (95% confidence interval: 0.712, 0.831) (Fig. 2).

## Discussion

Here, we harmonized large retrospective clinical data sets from five international cohorts with the aim to identify the best clinical discriminator(s) between sporadic bvFTD and late-onset PPDs. Three neuropsychological domains—global cognitive screening, letter fluency and attention—emerged as potential candidates in various logistic regression models, either when considered independently or simultaneously.

Our results showed the clinical importance of examining letter fluency in dementia, particularly when a differential diagnosis between bvFTD and PPD is considered. When diagnosis is uncertain, our study demonstrates that low letter fluency scores will favour bvFTD. Letter fluency task performance was the only test that was available in both groups as a measurement for executive functioning, and therefore, we were only able to investigate a very limited facet of executive function. As such, this finding may need to be taken with caution. Nevertheless, our finding contrasts with the late-onset frontal lobe study^47^—which shares some of the cases included in this study—where executive function tests did not differentiate between bvFTD and PPD.^48^ It is plausible that a low performance on letter fluency in the bvFTD group may have arisen from a breakdown in language skills, which can be present early in the disease course,^49^ rather than reflecting executive dysfunction. Taken together, although the discriminative utility of letter fluency seems promising, it requires, together with other (non-verbal) executive function tasks, further validation.

The global cognitive screening domain also emerged as a potential discriminator between bvFTD and PPD. This domain harmonized MMSE and ACE-III, instruments that both include tasks measuring a range of cognitive processes, such as orientation, language and memory. The similar nature of these instruments enabled us to combine them into a single domain. Importantly, the discriminating power of this domain was present even although the MMSE has previously shown limited sensitivity in distinguishing bvFTD from PPD in the late-onset frontal lobe study,^50^ and MMSE scores are often preserved in early FTD stages.^51^ The ACE-III has shown that it can differentiate FTD from Alzheimer’s disease dementia in mild dementia stages^52^ and from MDDs.^53^ In this context, this finding suggests that the discriminating power between bvFTD and PPD arises not from the components shared by the ACE-III and the MMSE, but from the additional cognitive tasks present in the ACE-III, namely semantic memory, language and visuospatial ability.^52,54^ Importantly, the letter fluency task is also included in the ACE-III. Whether ACE-III subdomains on their own, including letter fluency, contribute to the differentiation between bvFTD and PPD will be examined in the prospective DIPPA-FTD study.

The domain of attention emerged as another significant discriminator between the groups in the combined logistic regression model. Its contribution, however, was unexpected, as a worse attention test performance was associated with PPD, even though the PPD group scored significantly higher on attention than the bvFTD group. As discussed in greater detail below, it is plausible that this ‘inverted’ contribution may have arisen from the patient selection bias in the optimal model. Indeed, unlike in the global samples, the scores for the attention domain were identical in the bvFTD (0.62) and PPD (0.62) subgroups included in the optimal data set. An additional explanation is a potential influence of attention on the other cognitive domains. This position is supported with the highest AUC value being reached with the model that included attention, together with global cognitive screening, working memory and letter fluency. This suggests that it may be necessary to correct for attention performance in future analysis rather than incorporating it as isolated discriminator.

Separate logistic regression models carried out on each domain separately, to maximize information extraction from the data available, further demonstrated that self-reported presence of depressive symptoms gives a 7-fold higher odds of being in the PPD group. Almost three-quarters (71.4%) of PPD patients scored above the threshold for the presence of depressive symptoms compared with only a quarter (24.3%) in the bvFTD group. In addition, the magnitude of this association may be explained by the high frequency of MDD diagnosis in the PPD group and the lack of disease insight in the FTD group. Not enough data were available to include depressive symptoms in the optimal model, but these findings suggest it is essential to include this variable in future investigations. The facial emotion processing domain also emerged as a significant clinical discriminator between bvFTD and PPD in the single logistic regression models. This domain included the Facial Affect Selection Task and the Ekman-60 Faces; both tests have been proven to be sensitive instruments to detect social cognition disturbances.^30,55^ Impaired social cognition is one of the earliest and most distinctive features of bvFTD.^8^ As such, inclusion of social cognition tasks during the clinical workup is strongly recommended when diagnostically differentiating between bvFTD and PPD or between bvFTD and other neurological disorders.^9,56^

While shedding important light on the differential diagnosis between sporadic bvFTD and PPD, findings from this study need to be interpreted with caution. First, possible circularity cannot be entirely ruled out, since the tests included in the statistical analyses were also employed to classify the study participants. Moreover, most neuropsychological tests measure multiple cognitive processes, possibly increasing collinearity across the predictors. However, variance inflation factors across predictors in the most optimal model were smaller than or equal to 2 and were therefore considered low (Supplementary Fig. 5).

Second, this study relied on clinical judgment as the final diagnostic standard due to the absence of disease-specific biomarkers, underscoring the importance of studies like ours that aim to identify discriminative and diagnostic markers. While this reliance may introduce some degree of noise into the final diagnoses, steps were taken to minimize this risk. Specifically, only cases for which expert (bv)FTD clinicians were highly confident in the diagnosis were included, of which significant portion had follow-up, and individuals with possible bvFTD—known to have the highest diagnostic instability^13^—were excluded. Despite these meticulously chosen inclusion and exclusion criteria, it is possible that some PPD cases were not followed up long enough to rule out a conversion to bvFTD. Of note, in previous work, we found that between 1.9 and 3.7% of PPD cases changed to a bvFTD diagnosis over time, which would be equivalent to three to six individuals in this study. Similarly, the lack of systematic genetic screening beyond the three most common gene mutations raises the possibility of misclassification, with some sporadic cases potentially harbouring rare mutations or atypical FTD presentations being mistaken for PPD. However, our genetic testing criteria, combined with the use of the Goldman and Wood scores, significantly reduced this risk. Twenty patients included in the study were diagnosed with FTD or PPD despite displaying positive Alzheimer’s disease biomarkers. According to the clinical criteria,^2^ a diagnosis of probable bvFTD should only be made in the absence of biomarkers indicative of Alzheimer’s disease. Alzheimer’s disease co-pathology in bvFTD, however, is not uncommon and has been previously reported in up to 50% of patients.^57^ If anything, the rate of Alzheimer’s disease pathology in our bvFTD and PPD cohorts appears low. This is possibly explained by a hesitancy in diagnosing a patient with a psychiatric disorder in the presence of Alzheimer’s disease-positive biomarkers, even though the Alzheimer’s disease pathology has no clinical significant effect yet. We cannot entirely rule out whether or not the three PPD patients with Alzheimer’s disease-positive biomarkers in our study may have had prodromal Alzheimer’s disease symptoms. Importantly, additional analyses after excluding the 17 bvFTD and 3 PPD cases with positive Alzheimer’s disease biomarker did not change the results of this study.

Third, because of its retrospective design, the study relies on a convenience sample, which could reduce its novelty, and also results in high percentages of missing data. This issue is not uncommon when pooling data sets from different sources. Detailed information on neuropsychiatric symptoms was not available across all sites. Only apathy and depressive symptoms were consistently assessed, although using different measures. Other neuropsychiatric symptoms, such as anxiety and hallucinations, were not systematically assessed across all sites and could therefore not be harmonized. These missing data also limited direct comparisons across the neuropsychological and neuropsychiatric domains and prevented the inclusion of all nine domains in the final model. This is particularly relevant for the domains showing the highest ORs in the separate logistic regression models, namely, depressive symptoms and facial emotion processing. Moreover, the missing data required different harmonization methods across domains, which could theoretically influence the results. To address this potential issue, we performed a sensitivity analysis on the individual tests before applying harmonization methods (b) and (c). The analysis revealed similar differences between the groups and predictive values for each domain in the logistic regression models (Supplementary Tables 6 and 7). This consistency supports our findings despite the harmonization methods used. In addition, by applying multiple harmonization methods, we were able to maximize data extraction for comparisons, an approach that is useful when pooling retrospective data from rare and understudied populations. This study also demonstrates the challenges of harmonizing retrospective clinical data sets from different sites and the urgent need for a common prospective core protocol when investigating these patient populations.

Fourth, it is important to note that the relatively small number of participants in the PPD group (152 cases) compared with the sporadic bvFTD group (508 cases) may have limited the statistical power and generalizability of the findings. Moreover, the majority of the PPD group consisted of individuals with MDD (*n* = 107). This finding provides insight into the most likely underlying psychiatric disorder in a population presenting with behavioural changes after the age of 45 in a specialized FTD clinic. This may be explained by our inclusion criteria as the onset of a depressive episode after the age of 45 is more common later in life compared with conditions such as personality disorders or schizophrenia. However, the predominance of MDD in our PPD group potentially affects the generalizability of our findings to other PPDs. To address this limitation and provide more insights into the cognitive performance of the MDD group, we conducted a sub-analysis that included sporadic bvFTD and MDD cases only (Supplementary Material 8). This sub-analysis demonstrated that letter fluency emerged as the only significant predictor in the most optimal model for distinguishing MDD from sporadic bvFTD.

Lastly, the amount of data available, and therefore the number of patients, included in the optimal subset may have arisen from the level of diagnostic uncertainty in a patient. In other words, it is plausible that the greater the diagnostic doubt experienced by the clinician, the larger the number of investigations was carried out, resulting in more data from more domains. This data generation bias may have resulted in the optimal subset to comprise the more challenging patients, from a diagnostic perspective, across a larger number of domains considered important when in diagnostic doubt. This group, however, may well be a better representation of the early phase sporadic bvFTD during which symptoms are less pronounced and phenotypes more ambiguous. The primary aim of this study was to identify neuropsychological measures that can differentiate sporadic bvFTD from PPD. As a result, biomarkers such as neurofilament light or imaging data were not included in this analysis, even though their incorporation could enhance diagnostic accuracy. Future investigations, including the prospective design of DIPPA-FTD, aim to address this limitation by integrating biomarker data to complement and extend the findings presented here.^17^

The results from this study identified three cognitive measurements—letter fluency, global cognitive screening and attention—that have clinical relevance towards effectively distinguish sporadic bvFTD from late-onset PPD. Arguably, a diagnosis of a disorder as complex as bvFTD or PPD relies on the input of multiple features combined rather than single features. Nevertheless, our results support the clinical intuition that patients with bvFTD have lower performance on executive and global cognitive tests, while PPD patients have predominant attentional deficits. While the neuropsychiatric domain depressive symptoms and the facial emotion processing tests contained considerable missing data, they emerged as potential discriminators, warranting the need for further exploration in prospective studies.

## Supplementary Material

fcaf199_Supplementary_Data

## Data Availability

The data that support the findings of this study are available from the corresponding author, upon reasonable request. The code for this study is available via https://github.com/sterredeboer/DIPPA_braincomm.git.
