# Supplementary material for: Differentiating sporadic frontotemporal dementia from late-onset primary psychiatric disorders
Source: Brain Commun. 2025 May 22;7(3):fcaf199. doi: 10.1093/braincomms/fcaf199 (PMC12141780; doi:10.1093/braincomms/fcaf199)
Supplement: fcaf199_Supplementary_Data [file fcaf199_supplementary_data.pdf]

Supplementary material

S1. Distributions of diagnoses per DIPPA-FTD site

Supplementary Table 1. Distribution of diagnoses per DIPPA-FTD site.

SITE	FTD, N	PPD, N
Australia	100	21
Canada	0	6
Germany	157	31
Italy	81	20
The Netherlands	170	74

Abbreviations: FTD: frontotemporal dementia, PPD: primary psychiatric disorder.

S2. Neuropsychological and neuropsychiatric scores per PPD diagnosis.

Supplementary Table 2. Scores per domain within the PPD group

	Major depressive disorder n=107	Bipolar Disorder n=18	Psychiatry disorders NOS n=11	Schizophrenia /Schizoaffective disorder n=7	Delusional Disorder n=6	Manic episode n=2	OCD n=1
**Global cognitive screening, median (IQR)**	28.0 (3.0)	26.0 (3.0)	27.0 (4.0)	26.0 (2.25)	29.0 (0.75)	22.5 (6.5)	30 (n.a.)
**Facial emotion processing, median (IQR)**	0.8 (0.2)	0.7 (0.03)	0.96 (0.01)	0.7 (0)	n.a.	0.8 (n.a.)	n.a.
**Episodic memory, median (IQR)**	15.5 (8.)	19.0 (4.6)	18.8 (2.8)	n.a.	20.5 (0)	11 (0)	24 (0)
**Animal fluency, mean (SD)**	18.4 (6.1)	19.2 (2.8)	16.4 (6.7)	17.7 (4.7)	18.0 (6.1)	19.0 (n.a.)	15.0 (n.a.)
**Attention, mean (SD)**	0.61 (0.19)	0.55 (0.21)	0.67 (0.24)	0.54 (0.10)	0.43 (0.17)	0.41 (0.17)	0.48 (n.a.)
**Working memory, mean (SD)**	0.45 (0.19)	0.36 (0.19)	0.52 (0.29)	0.33 (0.21)	0.41 (0.18)	0.19 (0.07)	0.33 (n.a.)
**Executive function, median (IQR)**	10.8 (7.8)	11.0 (3.1)	12.0 (2.4)	6.8 (3.6)	13.0 (3.1)	11.7 (n.a.)	13.0 (n.a.)
**NPS – depressive symptoms, (present/absent)**	52/14	5/6	2/2	3/2	2/1	1/0	0/1
**NPS – apathy, (present/absent)**	27/6	4/5	4/0	2/0	1/2	1/1	1/0

Abbreviations: bvFTD: behavioral variant of frontotemporal dementia, PPD: primary psychiatric disorders, IQR: inter quartile range, NOS: not other specified, OCD: obsessive compulsive disorder, SD: standard deviation, NPS: neuropsychiatric symptoms.

S3. Group comparison of each clinical feature and output logistic regression per domain
3.1 Global Cognitive Screening

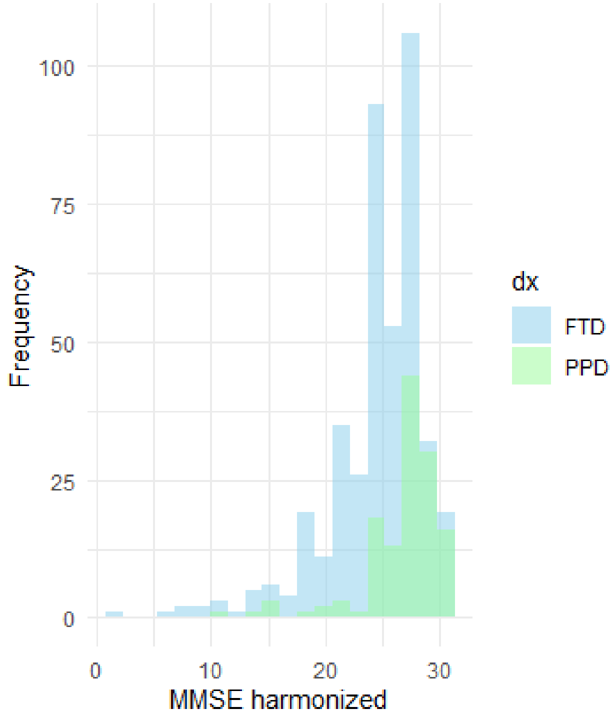

Supplementary Figure 3.1.1 Histogram of converted MMSE scores of subset of cohort (FTD N=419, PPD N=133)
Abbreviations: dx: Diagnsotic group. FTD: sporadic Frontotemporal Dementia. PPD: Primary Psychiatric Disorder. MMSE: Minimal Mental State Examination.

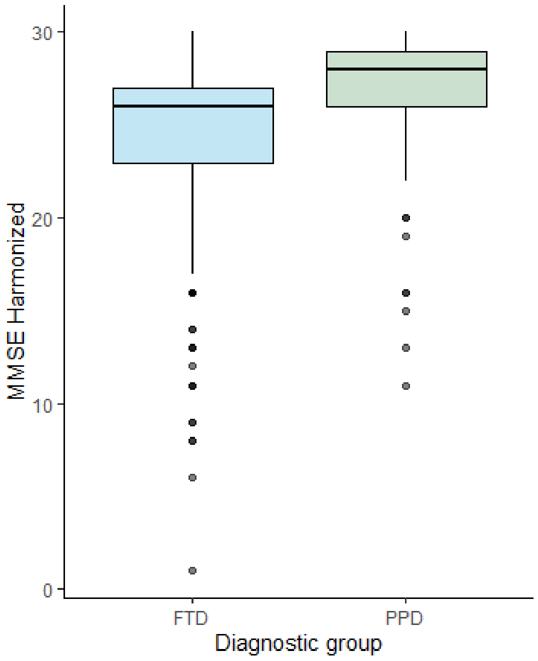

Supplementary Figure 3.1.2 Boxplot of converted MMSE score per diagnosis of subset of cohort (FTD N=419, PPD N=133). The PPD group had a significant higher score (median 28.0 (IQR 4.0)) compared to FTD (median 26.0 (IQR 4.0), Mann-Whitney U test $p<0.0001$).
Abbreviations: dx: Diagnsotic group. FTD: sporadic Frontotemporal Dementia. PPD: Primary Psychiatric Disorder. MMSE: Minimal Mental State Examination.

Supplementary Table 3.1.1 Logistic regression model of global cognitive screening of subset of cohort (FTD N=419, PPD N=133).

	Estimate	p	OR	[95% CI]
**(Intercept)**	**0.09**	**0.95**	**1.09**	**[0.07, 16.49]**
**Global cognitive Screening**	**0.21**	**<0.001**	**1.23**	**[1.14, 1.33]**
**Age**	**-0.09**	**<0.001**	**0.92**	**[0.89, 0.94]**
**Sex**	**0.46**	**0.04**	**1.59**	**[1.02, 2.51]**
**Education**	**-0.14**	**<0.001**	**0.87**	**[0.81, 0.94]**

Outcome: FTD (0), PPD (1)
Female (0), Male (1)
Corrected for: Age, Sex, Education
R2 Nagelkerke: 0.23
Abbreviations: OR: Odds Ratio, CI: confidence interval, FTD: sporadic Frontotemporal Dementia. PPD: Primary Psychiatric Disorder.

3.2 Facial Emotion Recognition

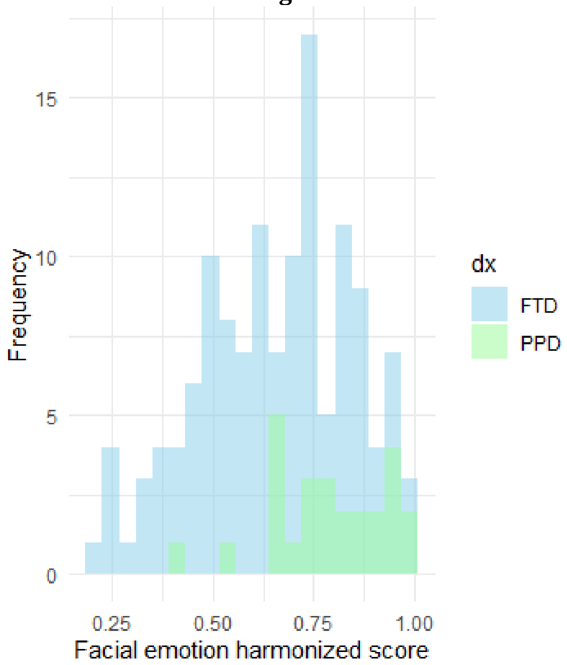

Supplementary Figure 3.2.1 Histogram of harmonized facial emotion recognition scores of subset of cohort (FTD N=132, PPD N=26)
Abbreviations: dx: Diagnsotic group. FTD: sporadic Frontotemporal Dementia. PPD: Primary Psychiatric Disorder.

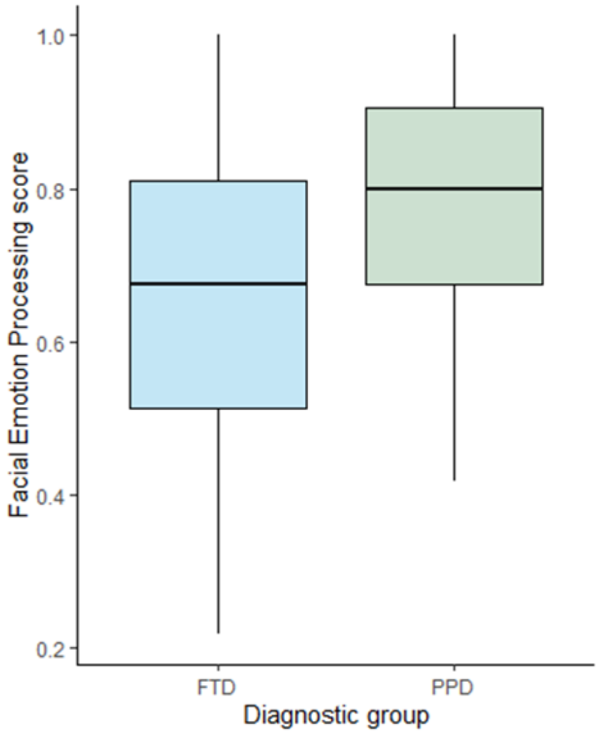

Supplementary Figure 3.2.2 Boxplot of Min-Max scaled Facial Emotion Recognition scores per diagnosis in subset of cohort (FTD N=132, PPD N=26). PPD scored significantly higher (median 0.80 (IQR 0.2)) compared to FTD (median 0.68 (IQR 0.3, Mann Whitney U-test p<0.01).

Supplementary Table 3.2.1 Logistic regression of Facial Emotion Recognition in subset of cohort (FTD N=132, PPD N=26).

	Estimate	p	OR	[95% CI]
(Intercept)	-0.85	0.72	0.43	[0.00, 47.56]
Facial Emotion Processing	0.52	<0.001	1.69	[1.26, 2.40]
Age	-0.04	0.13	0.96	[0.91, 1.01]
Sex	-0.05	0.91	0.95	[0.37, 2.56]
Education	-0.17	0.02	0.84	[0.73, 0.97]

Outcome: FTD (0), PPD (1)
Female (0), Male (1)
1 unit = 10% increase in score
R2 Nagelkerke: 0.20
Corrected for: Age, Sex, Education
Abbreviations: OR: Odds Ratio, CI: confidence interval, FTD: sporadic Frontotemporal Dementia. PPD: Primary Psychiatric Disorder.

3.3. Episodic Memory Score

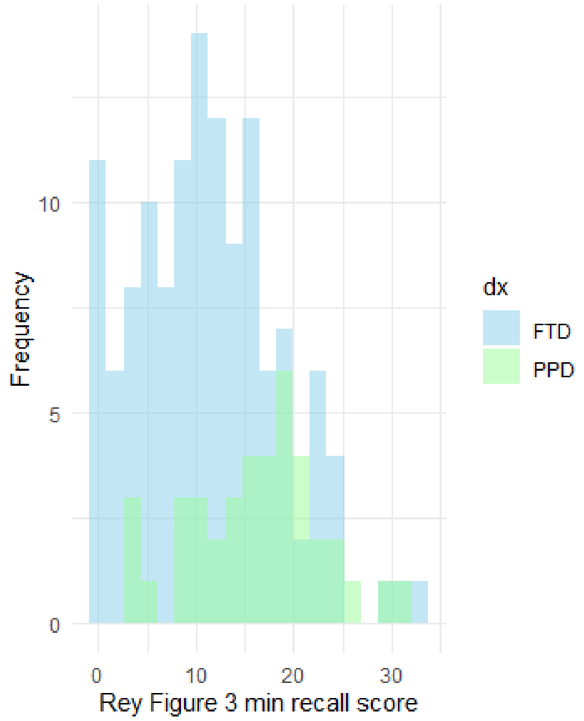

Supplementary Figure 3.3.1 Histogram of Rey Figure Recall scores of subset of cohort (FTD N=129, PPD N=40).
Abbreviations: dx: Diagnsotic group. FTD: sporadic Frontotemporal Dementia. PPD: Primary Psychiatric Disorder.

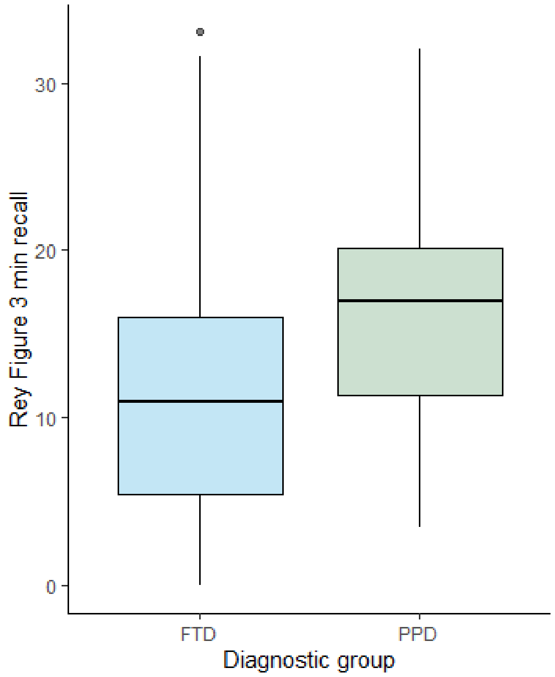

Supplementary Figure 3.3.2 Boxplot of Rey Figure Recall scores of subset of cohort (FTD N=129, PPD N=40).
PPD group scored (median 17.0 (IQR 8.7)) significantly higher than the FTD group (median 11.0 (IQR 10.5), Mann Whitney U-test p<0.0001).
Abbreviations: dx: Diagnsotic group. FTD: sporadic Frontotemporal Dementia. PPD: Primary Psychiatric Disorder.

3.4 Animal Fluency

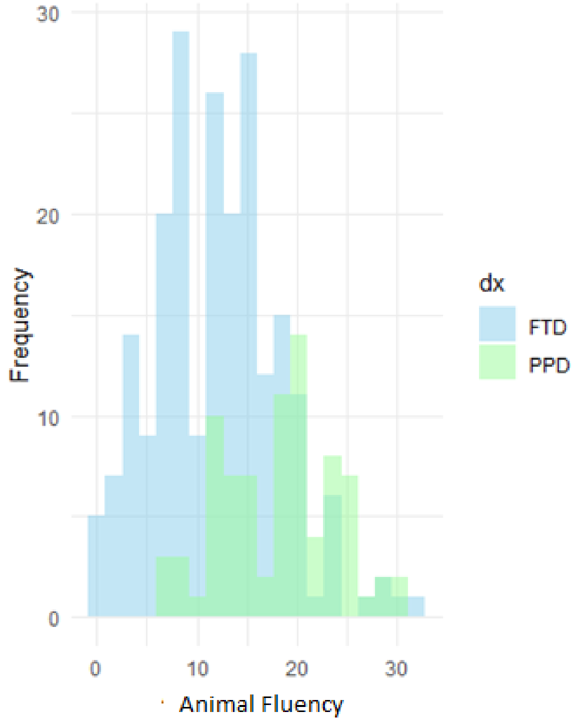

Supplementary Figure 3.4.1 Histogram of Animal Fluency of a subset of the cohort (FTD N=217, PPD N=82). Abbreviations: dx: Diagnsotic group. FTD: sporadic Frontotemporal Dementia. PPD: Primary Psychiatric Disorder.

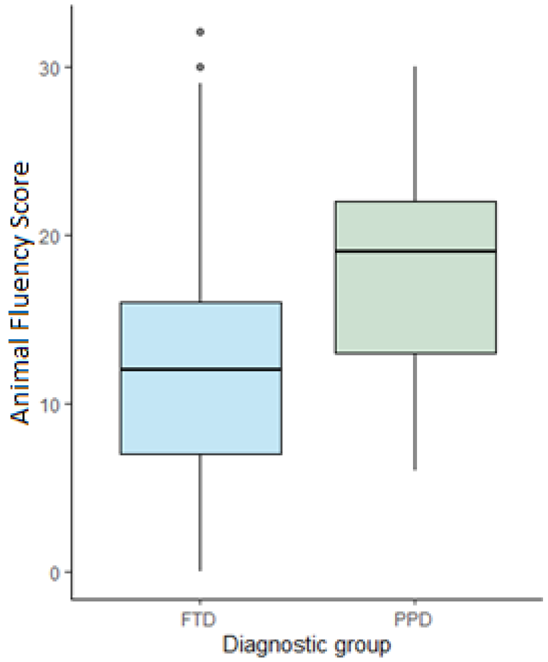

Supplementary Figure 3.4.2 Boxplot of Animal Fluency (y-axis) per diagnostic group of a subset of the cohort (FTD N=217, PPD N=82).
PPD scored significantly higher (mean 18.2 (SD 5.8)) compared to the FTD group (mean 11.9 (SD 6.3), T-test p<0.0001).
Abbreviations: FTD: sporadic Frontotemporal Dementia. PPD: Primary Psychiatric Disorder.

Supplementary Table 3.4.1 Logistic regression model of Animal Fluency in a subset of the cohort (FTD N=217, PPD N=82).

	Estimate	p	OR	[95% CI]
(Intercept)	0.38	0.79	1.47	[0.09, 24.87]
Animal Fluency	0.16	<0.001	1.17	[1.11, 1.23]
Age	-0.05	0.01	0.95	[0.92, 0.99]
Sex	0.12	0.70	1.13	[0.61, 2.10]
Education	-0.06	0.25	0.94	[0.84, 1.05]

Outcome: FTD (0), PPD (1)
Female (0), Male (1)
R^2 Nagelkerke: 0.28
Corrected for: Age, Sex, Education
Abbreviations: OR: Odds Ratio, CI: confidence interval, FTD: sporadic Frontotemporal Dementia. PPD: Primary Psychiatric Disorder.

3.5 Attention

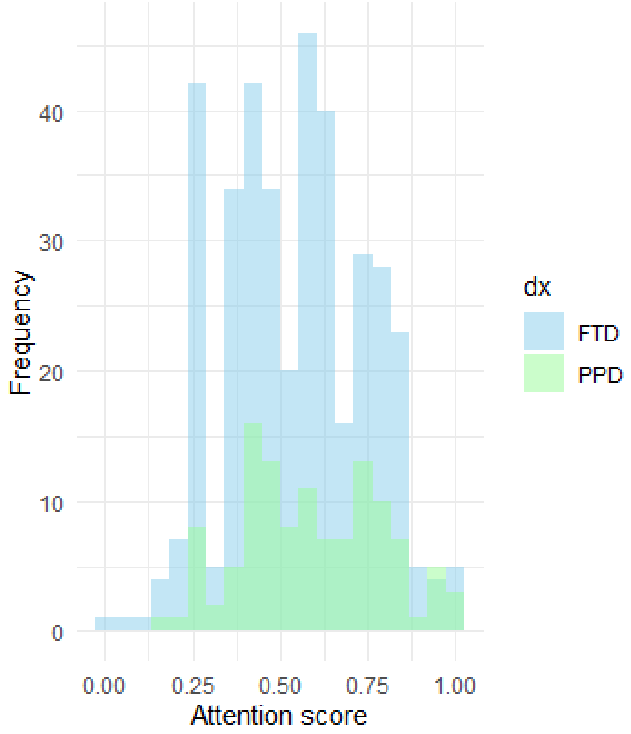

Supplementary Figure 3.5.1 Histogram of Min-Max scaled Attention Score in subset of cohort (FTD N=387, PPD N=118).
Abbreviations: dx: Diagnsotic group. FTD: sporadic Frontotemporal Dementia. PPD: Primary Psychiatric Disorder.

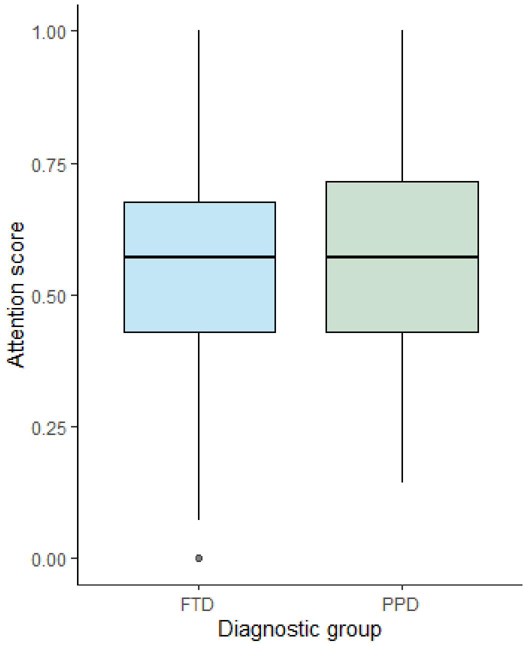

Supplementary Figure 3.5.2 Boxplot of Min-Max scaled Attention score in a subset of the cohort (FTD N=387, PPD N=118). There was no significant difference between the PPD and FTD group (FTD mean score 0.55 (SD 0.2), PPD mean score 0.59 (SD 0.2), T-test p=0.04).
Abbreviations: FTD: sporadic Frontotemporal Dementia. PPD: Primary Psychiatric Disorder.

Supplementary Table 3.5.1 Logistic regression of Attention Score in a subset of the cohort (FTD N=387, PPD N=118).

	Estimate	p	OR	[95% CI]
(Intercept)	4.74	0.00	114.55	[14.45, 987]
Attention score	0.09	0.13	1.10	[0.97, 1.24]
Age	-0.09	0.00	0.91	[0.89, 0.94]
Sex	0.28	0.22	1.33	[0.84, 2.12]
Education	-0.09	0.02	0.91	[0.84, 0.98]

Outcome: FTD (0), PPD (1)
Female (0), PPD (1)
R^2 Nagelkerke: 0.15
1 unit = 10% increase in score
Corrected for: Age, Sex, Education
Abbreviations: OR: Odds Ratio, CI: confidence interval, FTD: sporadic Frontotemporal Dementia. PPD: Primary Psychiatric Disorder.

3.6 Working Memory Score

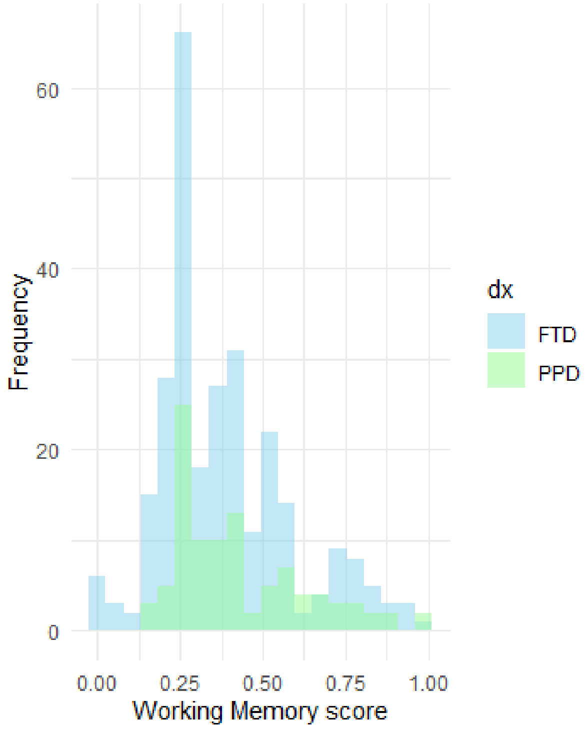

Supplementary Figure 3.6.1 Histogram of Min-Max scaled Working Memory Score in a subset of the cohort (FTD N=278, PPD =100).
Abbreviations: dx: Diagnsotic group. FTD: sporadic Frontotemporal Dementia. PPD: Primary Psychiatric Disorder.

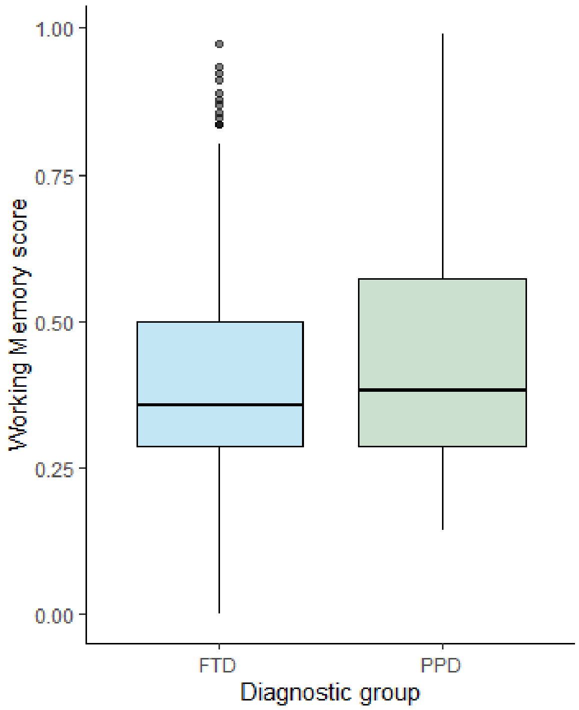

Supplementary Figure 3.6.2 Boxplot of Min-Max scaled Working Memory Score in a subset of the cohort (FTD N=278, PPD N=100). Scores did not significantly differ between groups (FTD mean working memory score 0.39 (SD 0.2) versus PPD mean score 0.43 (SD 0.2), T-test p=0.04)
Abbreviations: FTD: sporadic Frontotemporal Dementia. PPD: Primary Psychiatric Disorder.

Supplementary Table 3.6.1 Logistic regression of Working Memory Score in a subset of the cohort (FTD N=278, PPD N=100).

	Estimate	p	OR	[95% CI]
(Intercept)	3.81	0.00	45.14	[4.98, 447]
Working Memory	0.17	0.01	1.18	[1.05, 1.34]
Age	-0.07	<0.001	0.93	[0.90, 0.96]
Sex	0.28	0.27	1.33	[0.81, 2.22]
Education	-0.13	0.01	0.88	[0.80, 0.96]

Outcome: FTD (0), PPD (1)
Female (0),Male (1)
R^2 Nagelkerke: 0.11
1 unit = 10% increase in score
Corrected for: Age, Sex, Education
Abbreviations: OR: Odds Ratio, CI: confidence interval, FTD: sporadic Frontotemporal Dementia. PPD: Primary Psychiatric Disorder.

3.7 Letter Fluency

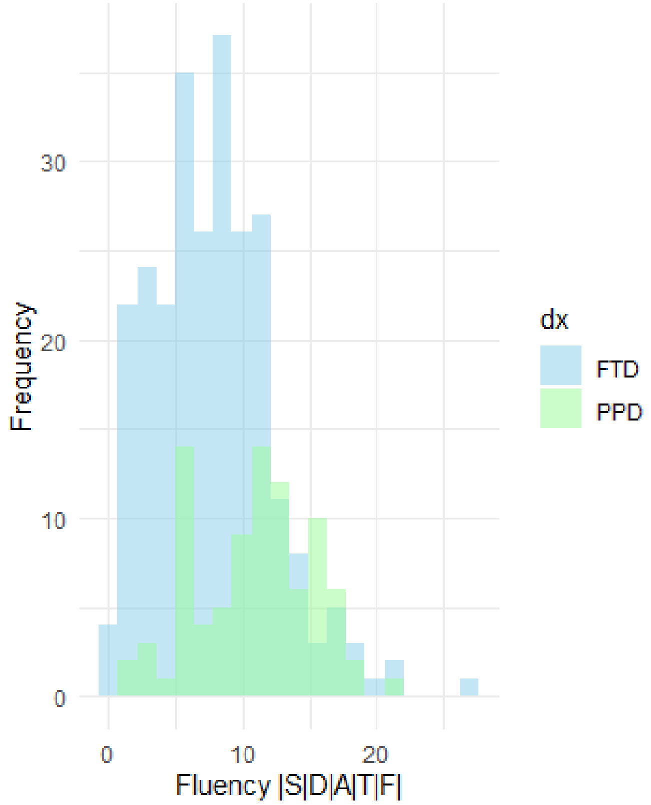

Supplementary Figure 3.7.1 Histogram of Letter Fluency in a subset of the cohort (FTD N=257, PPD=89). Abbreviations: dx: Diagnsotic group. FTD: sporadic Frontotemporal Dementia. PPD: Primary Psychiatric Disorder.

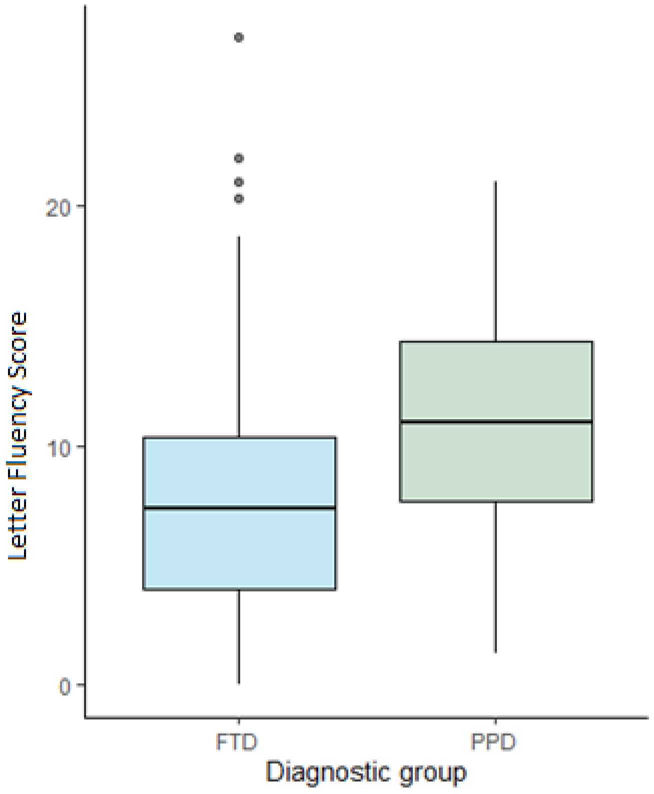

Supplementary Figure 3.7.2 Boxplot of Letter Fluency Score in a subset of the cohort (FTD N=257, PD N=89). PPD scored significantly higher (median 11.0 (IQR 6.7)) compared to the FTD group (median 7,3 (IQR 6.3), Mann Whitney-U test p<0.0001).
Abbreviations: FTD: sporadic Frontotemporal Dementia. PPD: Primary Psychiatric Disorder.

Supplementary Table 3.7.1 Logistic regression of Letter Fluency in a subset of the cohort (FTD N=257, PPD N=89).

	Estimate	p	OR	[95% CI]
(Intercept)	2.95	0.02	19.20	[1.67, 239.7]
Letter Fluency	0.16	<0.001	1.17	[1.11, 1.25]
Age	-0.06	<0.001	0.94	[0.91, 0.97]
Sex	0.12	0.68	1.12	[0.65, 1.96]
Education	-0.16	<0.001	0.85	[0.76, 0.95]

Outcome FTD (0), PPD (1)
Female (0), Male (1)
R^2 Nagelkerke: 0.20
Corrected for: Age, Sex, Education

Abbreviations: OR: Odds Ratio, CI: confidence interval, FTD: sporadic Frontotemporal Dementia. PPD: Primary Psychiatric Disorder.

3.8 Neuropsychiatric symptoms – Depressive Symptoms

Supplementary Table 3.8.1 Prevalence of depressive symptoms (present/absent)

Depressive symptoms	FTD n (%)	PPD n (%)	p-value (Chi-square analysis)
Present	34 (24.3)	65 (71.4)	<0.0001
Absent	106 (75.7)	26 28.6)	

Cut-off for absent and present was calculated using the Geriatric Depressions Scale (GDS ≥6 == Present), the Beck Depression Inventory II (BDI-II ≥14 == Present) and the Montgomery Åsberg Depression Rating Scale (MADRS ≥7 == Present).

Supplementary Table 3.8.2 Logistic regression of Neuropsychiatric symptoms – Depressive symptoms in a subset of the cohort (FTD N=140, PPD N=91)

	Estimate	p	OR	[95% CI]
(Intercept)	4.69	<0.001	108.79	[4.87, 2875]
Depressive symptoms	2.00	<0.001	7.41	[3.97, 14.27]
Age	-0.10	<0.001	0.91	[0.87, 0.95]
Sex	0.21	0.53	1.23	[0.65, 2.38]
Education	-0.01	0.82	0.99	[0.88, 1.11]

Outcome: FTD (0), PPD (1)
Female (0), Male (1)
R^2 Nagelkerke: 0.20
Corrected for: Age, Sex, Education
Abbreviations: OR: Odds Ratio, CI: confidence interval, FTD: sporadic Frontotemporal Dementia. PPD: Primary Psychiatric Disorder.

3.9 Neuropsychiatric symptoms – Apathy

Supplementary Table 3.9.1 Reported apathy (yes/no)

Reported Apathy	FTD n (%)	PPD n (%)	p-value (Chi-square analysis)
**Yes**	**85 (73.9)**	**40 (74.1)**	**1.00**
**No**	**30 (26.1)**	**14 (25.9)**	

Reported apathy was derived from the Starkstein Apathy rating ≥14 = Yes and the Neuropsychiatric Inventory (NPI apathy item level yes/no).

Supplementary Table 3.9.2 Logistic regression model in a subset of the cohort (FTD N=115, PPD N=54)

	Estimate	p	OR	[95% CI]
(Intercept)	6.65	<0.001	772.07	[20.31, 40358]
Apathy	-0.19	0.64	0.82	[0.37, 1.86]
Age	-0.11	<0.001	0.90	[0.85, 0.94]
Sex	1.02	0.01	2.78	[1.31, 6.21]
Education	-0.12	0.10	0.89	[0.77, 1.02]

Outcome: FTD (0), PPD (1)
Female (0), Male (1)
R^2 Nagelkerke: 0.21
Corrected for: Age, Sex, Education
Abbreviations: OR: Odds Ratio, CI: confidence interval, FTD: sporadic Frontotemporal Dementia. PPD: Primary Psychiatric Disorder.

3.10 Optimal Model

Supplementary Table 3.10.1 Logistic regression of optimal model in a subset of the cohort (N = 217 bvFTD, N = 75 PPD).

	Estimate	p-value	OR	[95% CI]
(Intercept)	-0.25	0.900	0.78	[0.01, 41.34]
Global cognitive screening	0.54	0.008	1.72	[1.18, 2.62]
Attention	-0.27	0.049	0.77	[0.58, 0.99]
Working memory	0.07	0.498	1.07	[0.87, 1.32]
Letter fluency	0.39	<0.001	1.47	[1.21, 1.80]
Age	-0.06	<0.001	0.94	[0.91, 0.98]
Education	-0.18	0.01	0.83	[0.73, 0.95]
Sex	0.22	0.48	1.25	[0.68, 2.34

Outcome: FTD (0), PPD (1)
Female (0), Male (1)
R^2 Nagelkerke: 0.23
Corrected for: Age, Sex, Education
Abbreviations: OR: Odds Ratio, CI: confidence interval, FTD: sporadic Frontotemporal Dementia. PPD: Primary Psychiatric Disorder

S4. PPD diagnosis included in optimal model

Supplementary Table 4. Breakdown of PPD diagnosis included in the optimal model.

PPD diagnosis	N
Major Depression disorder	55
Bipolar disorder	7
Psychiatric Disorders NOS	5
Delusional disorder	3
Schizophrenia	3
Manic episode	1
OCD	1

S5. Multicollinearity

Supplementary Figure 5 shows the variance inflation factor (VIF) for each of the included variables in the most optimal model.

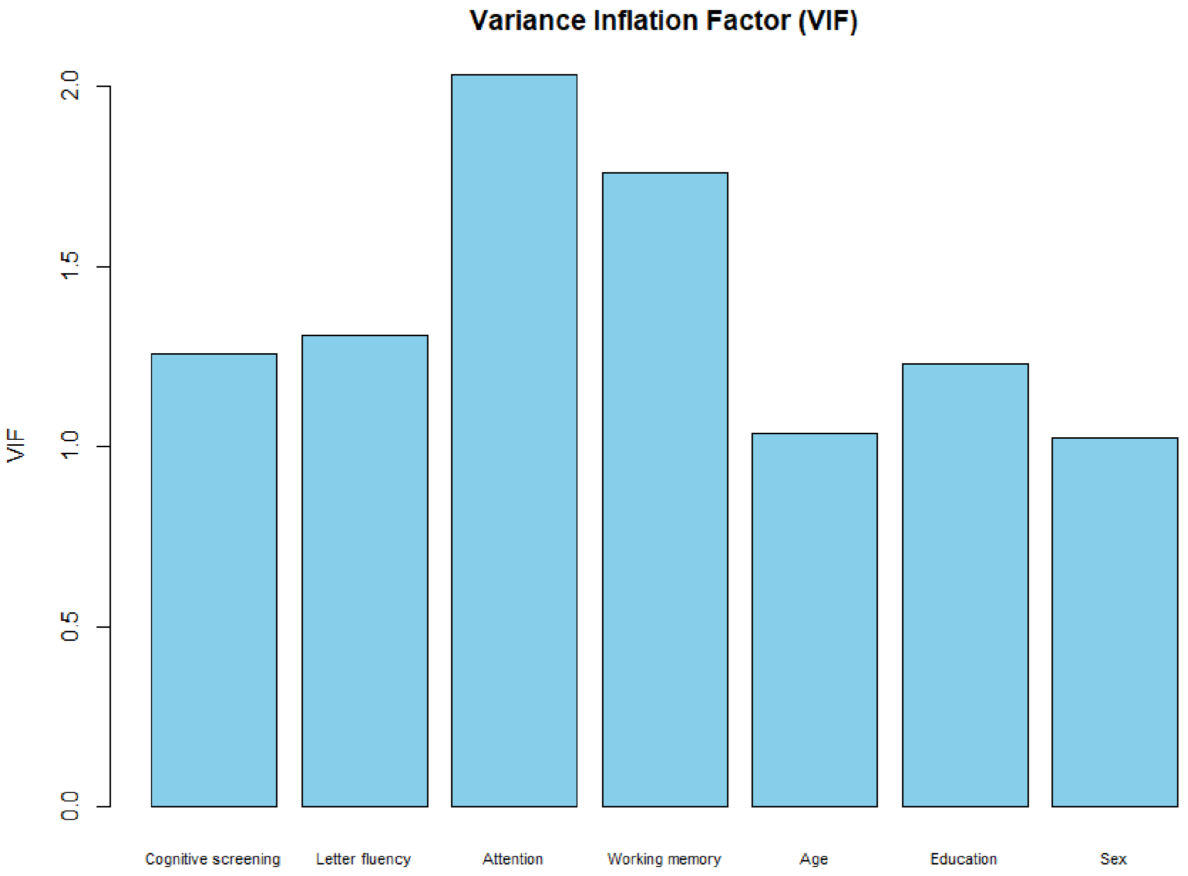

Supplementary Figure 5. Variance Inflation Factor for the most optimal model (N = 217 bvFTD, N = 75 PPD). VIF (y-axis) is given per variable (x-axis).
Abbreviations: VIF: variance inflation factor, bvFTD: behavioural variant frontotemporal dementia, PPD: primary psychiatric disorders.

S6. Test scores of each test prior to harmonization methods (b) and (c)

Supplementary Table 6. Test scores of tests prior to harmonization methods
[a]Mann-Whitney-U

	n FTD/PPD	Score FTD	Score PPD	p-value
**ACE-III, median (IQR)**	100/21	82.0 (14.9)	91.6 (6.1)	< 0.001[a]
**MMSE, median (IQR)**	329/117	25.0 (4.0)	28.0 (3.0)	<0.001[a]
**Ekman, mean (SD)**	50/18	31.5 (11.0)	44 (9.0)	<0.001[a]
**FAST, median (IQR)**	82/8	32.0 (8.0)	40.0 (2.3)	<0.001[a]
**Digit Span Forward, median (IQR)**	322/104	7.0 (3.7)	7.7 (4.0)	0.034[a]
**TMT-A, median (IQR)**	280/90	50.0 (34.3)	40.0 (28.8)	<0.001[a]
**Digit Span Backward, median (IQR)**	240/88	4.7 (2.7)	5.0 (2.17)	0.026[a]
**TMT-B minus TMT-A, median (IQR)**	208/82	72.0 (68.5)	53.5 (57.5)	0.002[a]

S7. Logistic regressions per test prior to harmonization methods (b) and (c)

Each logistic regression is corrected for: Age, Sex, Education. Outcome: FTD (0), PPD (1). Sex: Female (0), Male (1)

Supplementary Table 7.1 ACE-III.

	Estimate	p	Odds Ratio	[95% CI]
(Intercept)	-6.56	0.07	0.00	[0.00, 1.13]
ACE	0.11	<0.01	1.12	[1.05, 1.22]
Age	-0.06	0.07	0.95	[0.89, 1.00]
Sex	-0.41	0.50	0.67	[0.20, 2.27]
Education	-0.05	0.61	0.95	[0.76, 1.16]

Abbreviations: CI: confidence interval, ACE: Addenbrooke's Cognitive Examination-III

Supplementary Table 7.2. MMSE

	Estimate	p	Odds Ratio	[95% CI]
(Intercept)	1.19	0.43	3.28	[0.17, 59.74]
MMSE	0.20	<0.001	1.22	[1.13, 1.33]
Age	-0.10	<0.001	0.91	[0.88, 0.94]
Sex	0.61	0.02	1.84	[1.13, 3.04]
Education	-0.15	<0.001	0.86	[0.79, 0.93]

Abbreviations: CI: confidence interval, Mini-Mental State Examination

Supplementary Table 7.3. Ekman-60 Faces test

	Estimate	p	Odds Ratio	CI_lower
(Intercept)	-0.09	0.99	0.92	[0.00, 14950,47]
Ekman-60	0.23	<0.001	1.26	[1.13, 1.47]
Age	-0.11	0.05	0.90	[0.79, 0.99]
Sex	0.67	0.40	1.95	[0.42, 10.16]
Education	-0.26	0.03	0.77	[0.59, 0.95]

Abbreviations: CI: confidence interval.

Supplementary Table 7.4. FAST test

	Estimate	p	Odds Ratio	[95% CI]
(Intercept)	-19.31	0.02	4.10e-9	[4.46e-18, 0.01]
FAST	0.53	0.01	1.70	[1.23, 2.82]
Age	-0.12	0.10	0.89	[0.75, 1.01]
Sex	-0.59	0.60	0.55	[0.06, 5.92]
Education	0.39	0.10	1.47	[0.95, 2.44]

Abbreviations: CI: confidence interval, FAST: Facial Affect Selection Task.

Supplementary Table 7.5. Digit Span Forward test

	Estimate	p	Odds Ratio	[95% CI]
(Intercept)	4.64	<0.001	103.31	[10.76, 1091,62]
Digit Span Forward	0.07	0.17	1.07	[0.97, 1.18]
Age	-0.09	<0.001	0.92	[0.89, 0.94]
Sex	0.43	0.09	1.53	[0.94, 2.55]
Education	-0.10	0.02	0.91	[0.83, 0.98]

Abbreviations: CI: confidence interval.

Supplementary Table 7.6. TMT-A test

	Estimate	p	Odds Ratio	[95% CI]
(Intercept)	4.18	<0.001	65.14	[6.80, 694.22]
TMT-A	-0.01	0.03	0.99	[0.98, 1.00]
Age	-0.06	<0.001	0.94	[0.91, 0.97]
Sex	0.27	0.31	1.31	[0.78, 2.24]
Education	-0.08	0.10	0.92	[0.84, 1.01]

Abbreviations: CI: confidence interval, TMT: trail making test.

Supplementary Table 7.7. Digit Span Backward

	Estimate	p	Odds Ratio	[95% CI]
(Intercept)	3.62	<0.001	37.24	[3.28. 469.17]
Digit Span Backward	0.19	<0.001	1.21	[1.06, 1.38]
Age	-0.07	<0.001	0.93	[0.90, 0.96]
Sex	0.48	0.09	1.61	[0.93, 2.84]
Education	-0.14	<0.01	0.87	[0.78, 0.96]

Abbreviations: CI: confidence interval.

Supplementary Table 7.8. TMT-B minus TMT-A

	Estimate	p	Odds Ratio	[95% CI]
(Intercept)	3.72	<0.01	41.09	[3.63, 519.38]
TMT-B minus TMT-A	-0.01	0.02	0.99	[0.99, 1.00]
Age	-0.05	<0.001	0.95	[0.91, 0.98]
Sex	-0.04	0.90	0.96	[0.55, 1.70]
Education	-0.06	0.22	0.94	[0.85, 1.04]

Abbreviations: CI: confidence interval, TMT: trail making test.

S8. Sub-analysis of main analysis including only Major Depressive Disorder patients of the PPD group.

Supplementary Table 8.1 shows the demographics and average scores of each domain of the subset of the DIPPA cohort including sporadic and major depressive disorders.

Supplementary Table 8.1. Demographics and Performance on neuropsychological and neuropsychiatry domains in the sporadic bvFTD and MDD subset.

	**bvFTD (n) / PPD (n)**	**bvFTD**	**MDD**	**p-value**
N, % total	n.a.	508 (82.6)	107 (17.4)	n.a.
Sex, % male	n.a.	58.7	63.6	0.41[a]
Education (years) mean (SD)	n.a.	11.6 (3.3)	11.5 (3.2)	0.64[b]
Age at neuropsychological tests, median (IQR)	n.a.	65.4 (8.7)	59.2 (7.6)	<0.001[b]
Global cognitive screening, median (IQR)	419/96	26.0 (4.0)	28.0 (3.0)	<0.001[c]
Facial emotion processing, median (IQR)	132/17	0.68 (0.3)	0.80 (0.2)	<0.01[c]
Episodic memory score, median (IQR)	129/29	11.0 (10.5)	15.5 (8.0)	<0.01[c]
Animal fluency, mean (SD)	217/57	11.9 (6.3)	18.4 (6.1)	<0.001[b]
Attention, mean (SD)	387/78	0.55 (0.2)	0.61 (0.2)	0.01[b]
Working memory, mean (SD)	278/70	0.39 (0.2)	0.45 (0.2)	0.01[b]
Letter fluency, median (IQR)	257/64	7.3 (6.33)	10.8 (7.8)	<0.001[c]
NPS – depressive symptoms, (present/absent)	140/66	34/106	52/14	<0.001[a]
NPS – apathy, (present/absent)	115/33	85/30	27/6	0.48[a]

Abbreviations: bvFTD: behavioral variant of frontotemporal dementia, MDD: major depressive disorder, IQR: inter quartile range, SD: standard deviation, NPS: neuropsychiatric symptoms.

a. Chi-square

b. T-test

c. Mann Whitney U

Supplementary Table 8.2 shows the results of the logistic regression per domain in the MDD subset only including sporadic bvFTD and MDD of the DIPPA-FTD dataset.

Supplementary Table 8.2. Logistic regression of each domain per subset in sporadic bvFTD and MDD subset.

	Estimate	p	Odds Ratio	[95% CI]
Global Cognitive screening	0.22	<0.001	1.24	[1.14, 1.37]
Facial emotion processing	0.58	<0.01	1.79	[1.25, 2.78]
Episodic memory score	0.07	0.02	1.07	[1.01, 1.14]
Animal fluency	0.15	<0.001	1.16	[1.10, 1.23]
Attention score	0.16	0.03	1.17	[1.02, 1.36]
Working memory	0.20	<0.01	1.22	[1.06, 1.40]
Letter fluency	0.15	<0.001	1.17	[1.09, 1.25]
NPS - depressive symptoms	2.41	<0.001	11.08	[5.38, 24.18]
NPS - apathy	0.15	0.79	1.16	[0.42, 3.57]

Abbreviations: CI: confidence interval, NPS: neuropsychiatric symptoms, OR: odds ratio. Reference group is bvFTD. All regression analyses are corrected for age, sex and education. Each row represents a separate logistic regression analysis in a subset of the total cohort. See Table 3 column 3 for number of cases included in each model. See supplementary material S2 for output of all separate models.

As a final analysis in the MDD sub-analysis, we re-run the most optimal model (bvFTD n=217, MDD n=55) including the variables Global Cognitive screening, Attention score, Working memory, Letter fluency, Age, Sex and Education.

Of the neuropsychological domains, letter fluency was the only significant predictor (p=0.001, see Supplementary Table 8.3). The significant model gave an AUC of 0.766 (see Supplementary Figure 8).

Supplementary Table 8.3. Logistic regression model of optimal dataset in the sporadic bvFTD and MDD subset.

	Estimate	p	Odds Ratio	[95% CI]
(Intercept)	0.41	0.86	1.51	[0.01, 124.54]
Global cognitive screening	0.42	0.06	1.52	[1.01, 2.41]
Attention	-0.11	0.44	0.89	[0.67, 1.18]
Working memory score	0.06	0.58	1.06	[0.85, 1.32]
Letter fluency	0.35	0.001	1.41	[1.15, 1.75]

Abbreviations: CI: confidence interval, OR: odds ratio. bvFTD: behavioral variant of frontotemporal dementia. PPD: primary psychiatric disorders.
Corrected for age, education and sex. All variables are min-max scaled. One unit increase represents 10% increase in higher score for each variable. Reference group: MDD (0), sporadic bvFTD (1).

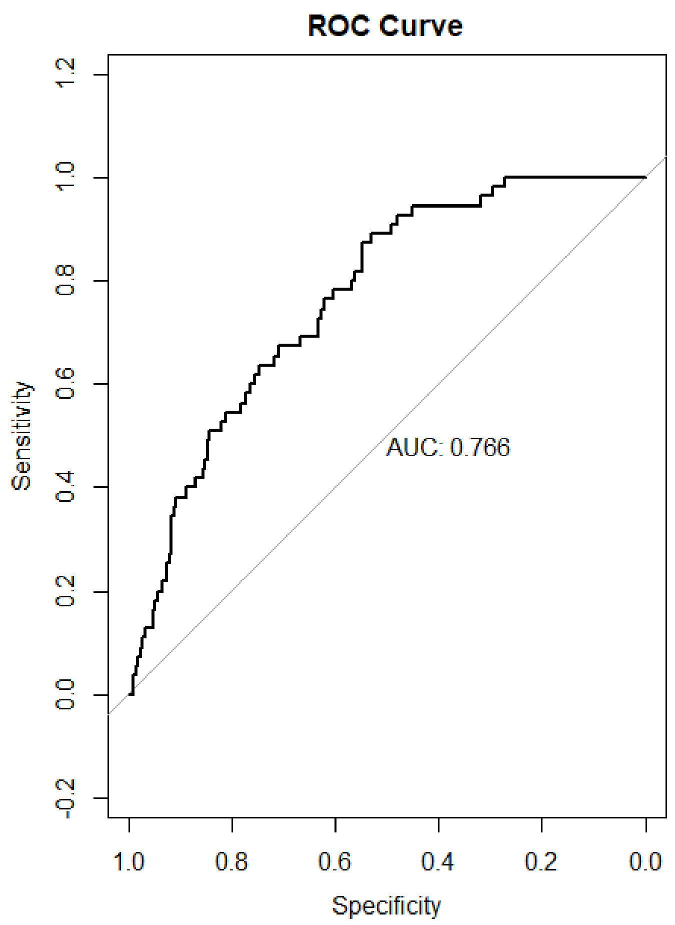

Supplementary Figure 8. ROC-curve from most optimal model in the sporadic bvFTD vs. MDD subset (bvFTD n=217, MDD n=55).
Abbreviations: ROC-Curve: receiver operating characteristic curve, AUC: area under the curve, bvFTD: behavioural variant of frontotemporal dementia. MDD: major depression disorder.

Supplementary Methods

M1. Correlations harmonized tests

In a subset of cases where both tests were administered and subsequently harmonized into a unified metric for the purposes of this study, we conducted a correlation analysis to assess the relationship between the two tests. This examination aimed to validate the harmonization methods employed for domain ii, v and vi.

(ii) Facial Emotion Processing

Within the FRONTIER cohort (Sydney, Australia), a total of n=290 cases were administered both the Ekman-60 and the FAST. A breakdown of the diagnostic groups of the n=290 cases can be found in Table M1. Results of correlation tests between the Ekman-60 total score and the FAST total score in the overall patient group and per (relevant) diagnostic group can be found in Table M2.

Supplementary Table M1. Diagnostic groups within FRONTIER cohort having both the Ekman-60 and FAST test administered.

Diagnostic group	n
Healthy Controls	66
Alzheimer's Disease	71
bvFTD	53
CBD	22
nfvPPA	18
SD left	17
PSP	14
SD right	8
FTD-MND	7
MCI	5
Organic other	4
Psychiatric disorder	3
DLB	2
Vascular Dementia	2
Aphasic other	1
Non-progressor FTD	1
Parkinon's Disease	1
TBI	1

Abbreviations: bvFTD: behavioural variant frontotemporal dementia, CBD: cortisobasal degeneration, nfvPPA: nonfluent variant primary progressive aphasia, SD: semantic dementia, PSP: progressive supranuclear palsy, FTD-MND: frontotemporal dementia motor neuron disease, MCI: mild cognitive impairment, DLB: dementia lewy body, TBI: traumatic brain injury.

Supplementary Table M2. Correlation between Ekman-60 test and FAST total score in each diagnostic group

Diagnostic group	Spearman r	p-value
**Control**	0.621	<0.001
**All Patients**	0.740	<0.001
**bvFTD**	0.737	<0.001
**nfvPPA**	0.692	0.001
**SD Left**	0.585	0.022
**SD Right**	0.790	0.020
**Alzheimer's Disease**	0.737	<0.001
**lvPPA**	0.839	<0.001

Abbreviations: bvFTD: behavioural variant frontotemporal dementia, CBD: cortisobasal degeneration, nfvPPA: nonfluent variant primary progressive aphasia, SD: semantic dementia, PSP: progressive supranuclear palsy, FTD-MND: frontotemporal dementia motor neuron disease, MCI: mild cognitive impairment, DLB: dementia lewy body, TBI: traumatic brain injury.

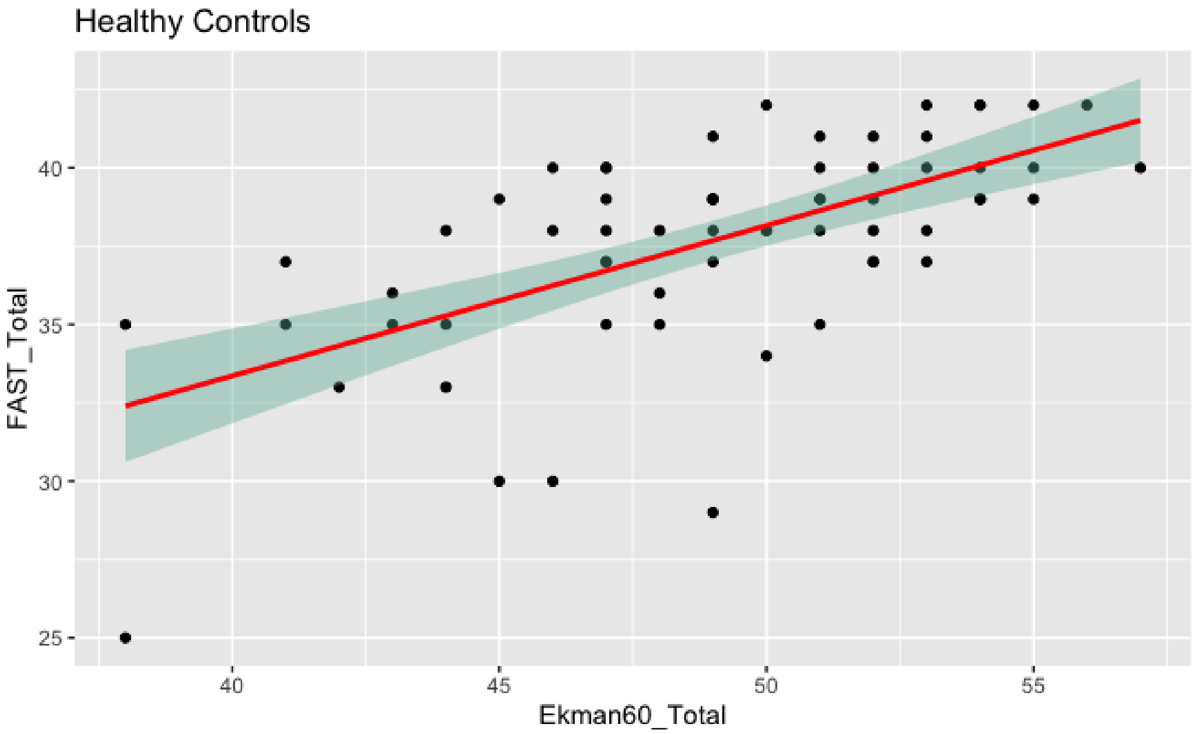

Supplementary Figure M1. Correlation between FAST Total score (y-axis) and Ekman-60 total score (x-axis) within Healthy Control (n=66, spearman r=0.621, p-value <0.001).

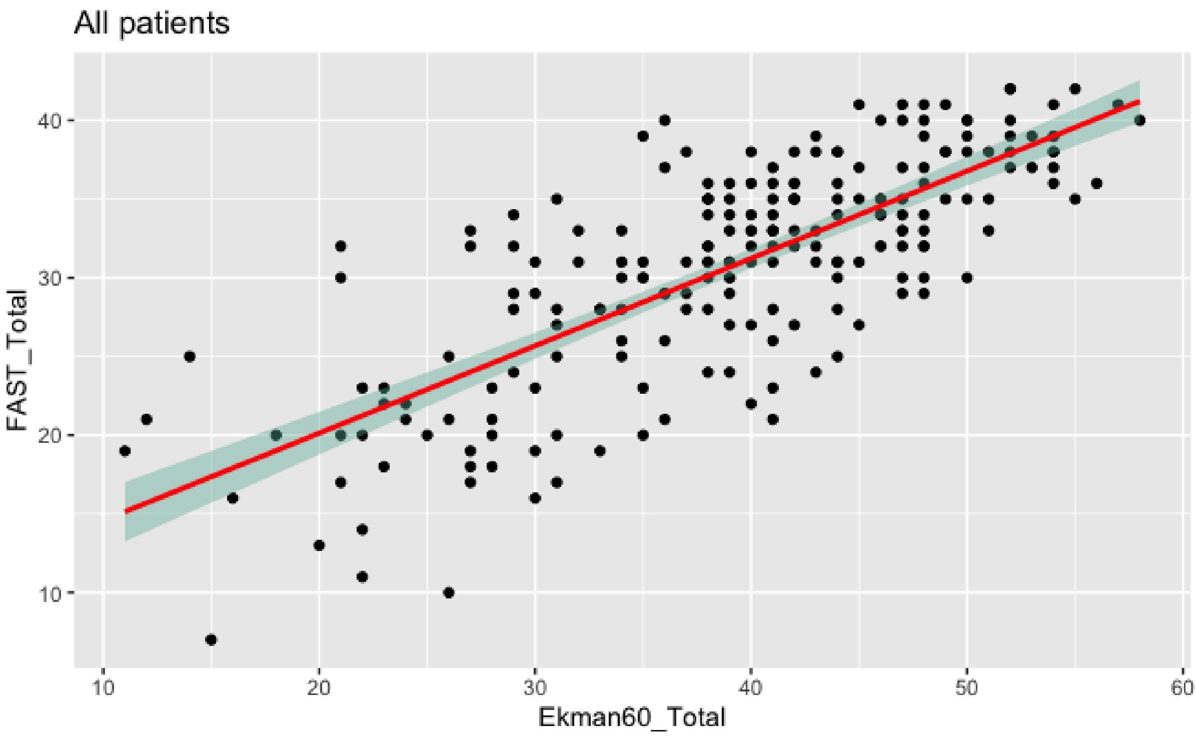

Supplementary Figure M2. Correlation between FAST Total score (y-axis) and Ekman-60 total score (x-axis) within All Patients (n=224, spearman r=0.740, p-value <0.001)

<u>(v) Attention score</u>

In the entire DIPPA-FTD retrospective cohort n=292 (213 bvFTD and 79 PPD) had both the TMT-A and Digit Span Forward administered. Supplementary Figure M4 shows the correlation between the TMT-A and the Digit Span Forward score. Spearman r=-0.25, p-value <0.001. If a case, after removal of the outliers and Min-Max scoring, had both TMT-A and Digit Span Forward test administered, the value of the Digit Span Forward test was used for the harmonized Attention score.

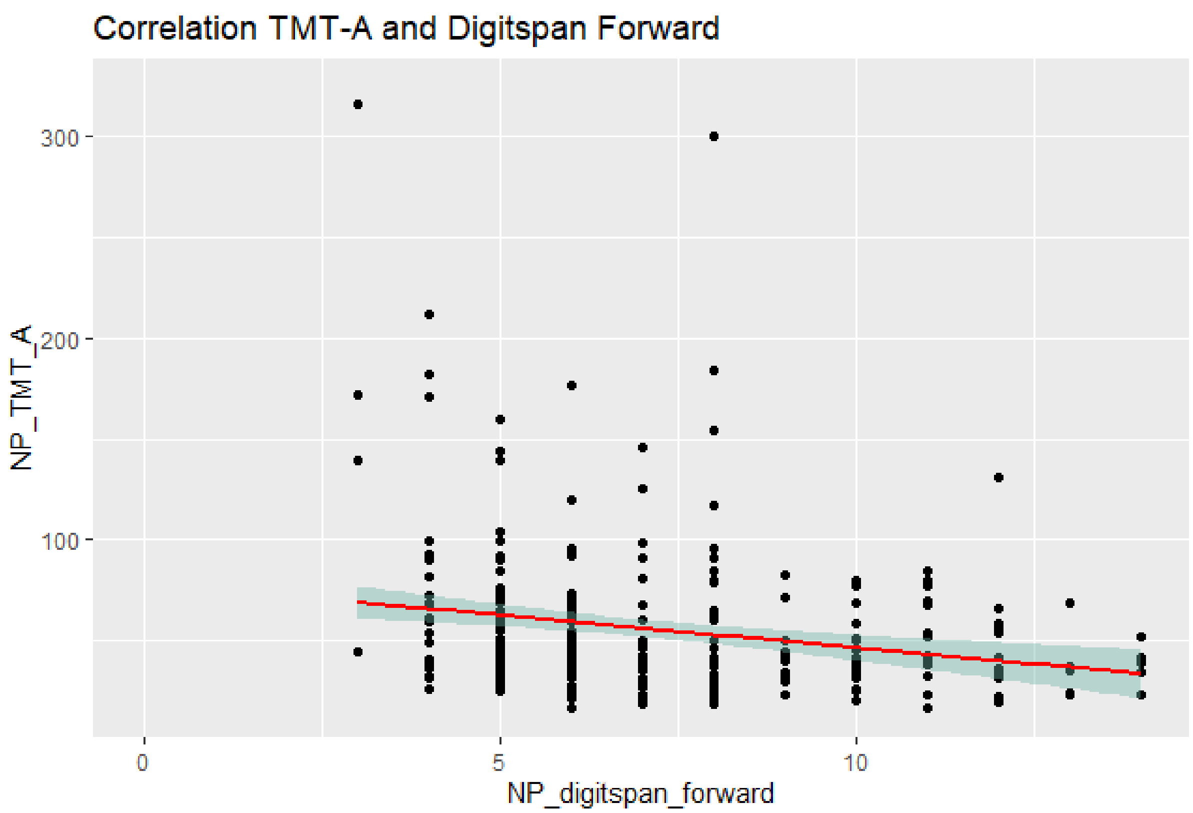

Supplementary Figure M3. Correlation between TMT-A score (y-axis) and Digit Span Forward score (x-axis) in the DIPPA-FTD cohort. In the entire DIPPA-FTD retrospective cohort n=292 (213 bvFTD and 79 PPD) had both the TMT-A and Digit Span Forward administered. Supplementary Figure M4 shows the correlation between the TMT-A and the Digit Span Forward score. Spearman r=-0.25, p-value <0.001. If a case, after removal of the outliers and Min-Max scoring, had both TMT-A and Digit Span Forward test administered, the value of the Digit Span Forward test was used for the harmonized Attention score.

(vi) Working Memory

Within the DIPPA-FTD retrospective cohort, n=146 had both the TMT-B and the Digit Span Backward administered. Supplementary Figure M5 shows the correlation between the TMT-B (minus TMT-A) score and Digit Span Backward score in the DIPPA-FTD cohort. Spearman r = -0.24, p-value <0.001. If a case, after removal of outliers and Min-Max scaling, had both the Digit Span Backward and TMT-B – TMT-A score available, the Digit Span Backward score was used for the harmonized Working Memory score.

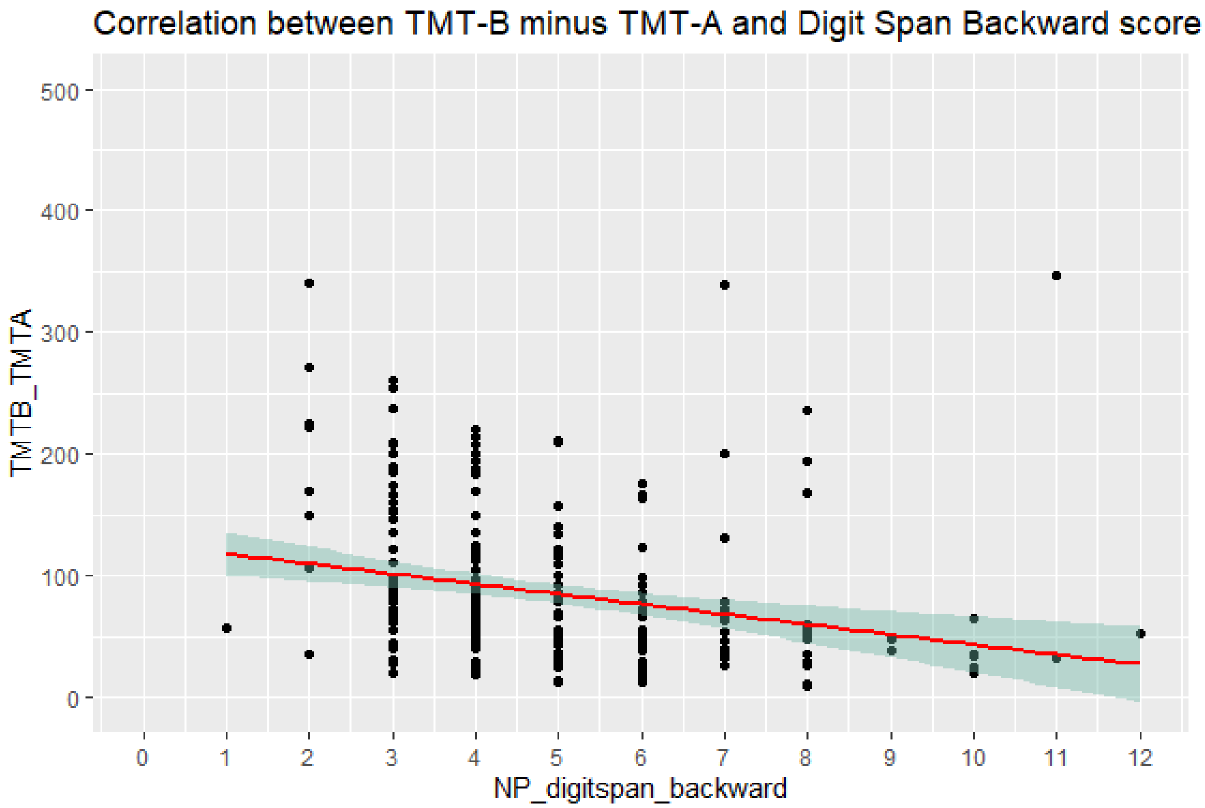

Supplementary Figure M4. Correlation between TMT-B (minus TMT-A) (y-axis) and Digit Span Backward score (x-axis) in the DIPPA-FTD cohort. Within the DIPPA-FTD retrospective cohort, n=146 had both the TMT-B and the Digit Span Backward administered. Supplementary Figure M5 shows the correlation between the TMT-B (minus TMT-A) score and Digit Span Backward score in the DIPPA-FTD cohort. Spearman r = -0.24, p-value <0.001. If a case, after removal of outliers and Min-Max scaling, had both the Digit Span Backward and TMT-B – TMT-A score available, the Digit Span Backward score was used for the harmonized Working Memory score.

S9. Difference in letter fluency within each site

Supplementary Table 9. Difference in letter fluency scores between bvFTD and PPD per site.

	**bvFTD**	**PPD**	**p-value[a]**
Australia (n=102), letter fluency median (IQR)	9.2 (5.9)	10.2 (4.1)	<0.001
Canada (n=3), letter fluency median (IQR)	16.0 (2.3)	n.a.	n.a.
Germany (n=81), letter fluency median (IQR)	8.0 (7.0)	6.5 (6.0)	0.02
Netherlands (n=160), letter fluency median (IQR)	7.0 (5.5)	10.3 (6.7)	<0.001

Abbreviations: bvFTD: behavioral variant of frontotemporal dementia, PPD: primary psychiatric disorders, IQR: inter quartile range.
[a] Mann Whitney U test
